# Consensus based SoC trajectory tracking control design for economic-dispatched distributed battery energy storage system

**DOI:** 10.1371/journal.pone.0232638

**Published:** 2020-05-14

**Authors:** Shafaat Ullah, Laiq Khan, Rabiah Badar, Ameen Ullah, Fazal Wahab Karam, Zain Ahmad Khan, Atiq Ur Rehman

**Affiliations:** 1 Department of Electrical and Computer Engineering, COMSATS University Islamabad, Abbottabad Campus, Abbottabad, KP, Pakistan; 2 Department of Electrical and Computer Engineering, COMSATS University Islamabad, Islamabad, Pakistan; Beijing Institute of Technology, CHINA

## Abstract

The state-of-charge (SoC) of an energy storage system (ESS) should be kept in a certain safe range for ensuring its state-of-health (SoH) as well as higher efficiency. This procedure maximizes the power capacity of the ESSs all the times. Furthermore, economic load dispatch (ELD) is implemented to allocate power among various ESSs, with the aim of fully meeting the load demand and reducing the total operating cost. In this research article, a distributed multi-agent consensus based control algorithm is proposed for multiple battery energy storage systems (BESSs), operating in a microgrid (MG), for fulfilling several objectives, including: SoC trajectories tracking control, economic load dispatch, active and reactive power sharing control, and voltage and frequency regulation (using the leader-follower consensus approach). The proposed algorithm considers the hierarchical control structure of the BESSs and the frequency/voltage droop controllers with limited information exchange among the BESSs. It embodies both self and communication time-delays, and achieves its objectives along with offering plug-and-play capability and robustness against communication link failure. Matlab/Simulink platform is used to test and validate the performance of the proposed algorithm under load disturbances through extensive simulations carried out on a modified IEEE 57-bus system. A detailed comparative analysis of the proposed distributed control strategy is carried out with the distributed PI-based conventional control strategy for demonstrating its superior performance.

## 1 Introduction

The main trend in electric power system, for the past few decades, has been the modification of conventional power system. This trend was triggered by several aspects including environmental, technological and economic aspects. The idea was to incorporate smaller distributed generating units (DGUs) into existing power system so as to mitigate the climate change concerns and the increasing energy demand. This led to the development of the so-called microgrid (MG), having the ability of wide integration of renewable energy sources (RESs). The MGs are low voltage (LV) electricity networks consisting of DG units, energy storage system (ESS), loads and interconnecting power lines [1].

The frequently varying load demands and the intermittent nature of the RESs cause a supply-demand imbalance in the MG. This in turn causes the frequency of the MG to vary because of its low inertia. To deal with this problem, ESSs (such as batteries) are installed and operated in a MG. They can be exploited as a buffer to mitigate the active power imbalance, by absorbing excessive power during off-peak load periods (peak generation periods) and releasing the same during peak-load periods (off-peak generation periods) [2].

ESSs are flexible power electronic-based devices, having a fast response time, that enhance the power quality and reliability of the MGs by ensuring an uninterrupted supply of power. They act as fundamental back-up supply during autonomous mode of the MG, by providing grid-forming generation, and black-start when the MG is reconnected to the utility grid. Furthermore, ESS provides the MG with several other advantages, including: improved efficiency, peak shaving and congestion alleviation. Due to high prices of the batteries, an ESS is still among the most expensive components in a MG. To prolong its life cycle, an ESS needs a special attention for its management. For example, the state-of-charge (SoC) of ESS should be kept in a certain safe range (20% to 80%) for ensuring its state-of-health (SoH) as well as higher efficiency. The SoC level of a battery actually depends on its specific production technology as well as operational history. In a system of multiple ESSs, it is often desired that all the SoC levels remain synchronized/balanced, such that, no single unit is permitted to charge/discharge more than the others. This procedure maximizes the power capacity of the ESSs all the times. An ESS tending to go outside its safe SoC range is immediately forced to disconnect for prevention of the possible damage [3, 4]. Due to the internal battery resistance, the charging efficiency of a battery energy storage system (BESS) is dependent on the charging rate (charging current), as reported in [5, 6]. Lower the charging rate, higher will be the charging efficiency, and vice versa. Charging efficiency exhibits certain dependence on current SoC, as well. It is generally believed that the charging efficiency is higher (over 95%) at lower SoC, and lower at higher SoC (top-of-charge) [7]. The impact of this lower charging efficiency at higher SOC would be a considerable reduction in actual available stored energy. Because, almost half the available energy is going to produce losses, instead of charging the battery.

The installation of an inappropriate or very large size ESS can result in energy wastage as well as increased cost of the system. Therefore, the energy management of an ESS is essential for proper control and operation of the MG. Centralized control strategies have been used for many years for managing the operation of ESS in a MG. However, they require a complex communication network and global information of all the nodes in the system, which is not only difficult to gather in time, but may also pose single-point-of-failure (SPOF) problem. The SPOF means that with the failure of the single (centralized control) unit, the whole control system fails down. Compared with the conventional centralized control strategy, a distributed cooperative control strategy, in which agents are connected through a sparse communication network, have been found more robust, reliable, economical, scalable and flexible. Because, only neighboring nodes are required to share information locally, thus avoiding a heavy communication load at the central control unit, as found in a centralized control strategy [8, 9]. The term distributed implies that the controller needs a communication network through which each agent (i.e. energy node or DG) can receive information of its neighboring agents only. While the term cooperative implies that, in contrast to the competitive control, all the agents act as single group to achieve a common synchronization goal by taking cooperative decisions [10].

Distributed cooperative control of multi-agent systems is mainly categorized into the leaderless consensus (or regulator synchronization problem) and the leader-follower consensus (or tracking synchronization problem). In the leaderless consensus, all the nodes of the MG synchronize to a common value that is not prescribed or controllable and depends on initial conditions. While in the leader-follower consensus, a leader or control node (one of the networked nodes) acts as a command generator by dictating the desired value. The rest of the nodes (i.e. followers) synchronize their outputs with that of the leader. The neighboring nodes can communicate with each other. Moreover, the leader node is only connected to a small portion of the total networked nodes [10, 11].

### 1.1 Related work

Consensus based control of the BESSs is fairly a new research area introduced in the literature recently. Compared with SoC balancing of the BESSs, as reported in [12–14], the modern consensus designs also address other issues of the BESSs, including regulation of frequency and voltage, synchronization of energy levels as well as active and reactive power sharing using exchange of information among the neighboring BESSs [15]. In [16], the authors have proposed a distributed consensus based algorithm for SoC balancing of the grid-connected BESSs consisting of 20 battery packages. However, the convergence speed of the algorithm was too slow, that is, around 2 hours. Furthermore, the droop controllers for frequency and voltage regulation have not been considered. A relatively simple and better linear quadratic regulator (LQR) based consensus algorithm was proposed in [17] for synchronizing the SoC as well as the power levels of the BESSs, with limited information exchange. The proposed design operated effectively both in the islanded and grid-connected mode. However, not only the convergence speed of the algorithm was very slow, that is, around 30 minutes, but also the droop controllers for frequency and voltage regulation have been ignored. Another LQR-based consensus algorithm was proposed in [18], similar to the one proposed in [17], for dynamic energy level balancing of the BESSs in droop controlled MGs. Detailed small signal battery dynamics have been considered in the design, however, the convergence speed was very slow, that is, around 1 hour. In [19], a novel nonlinear sliding mode control (SMC) based multi-agent consensus algorithm was proposed for SoC balancing of the distributed BESSs in a DC MG. This nonlinear consensus based strategy offered some qualitative improvements over the existing linear consensus based SoC balancing strategies in terms of avoiding circulating currents and providing plug-and-play capabilities. However, the algorithm has a very slow convergence speed by developing consensus in around 40 minutes.

### 1.2 Motivation and contributions

The authors, in [15], have proposed a novel and very remarkable distributed control design based on consensus theory for proportional active and reactive power sharing, synchronization of energy levels, and frequency and voltage regulation of multiple BESSs. Self and communication time-delays have been explicitly considered in the design. The proposed consensus algorithm has a very fast convergence speed, by developing consensus approximately within 10 seconds, because of independent controls of each of power and energy levels, active and reactive powers, frequency and voltage regulation, with and without time-delays. However, this algorithm has not been tested for plug-and-play capabilities and robustness against communication link failure. This was the main driving force that motivated the authors to undertake this research work and move further in this area by putting their own contributions. The authors, in this research article, have proposed a consensus based SoC trajectory tracking control design for economic-dispatched distributed BESSs in a MG. The MG itself is considered as a multi-agent system with the BESSs serving as agents, where each BESS can communicate with its neighboring BESSs through a sparse communication network. Without requiring any centralized controller, the proposed consensus based distributed algorithm in this article has been applied to multiple BESSs for achieving SoC trajectory tracking control, active and reactive power sharing control, voltage and frequency regulation as well as dispatching the load economically.

In order to efficiently operate the BESSs in a smart grid, this article proposes a time-delay resilient leader-follower consensus based fully distributed control strategy for fulfilling multiple objectives. The significant contributions of this research work, that distinguish it from the current literature, are summarized as follows:

1. The proposed distributed multi-agent consensus control algorithm tracks the BESSs SoC reference trajectories, generated on the basis of time of use (ToU) pricing, for 24 hours varying load.
2. The proposed algorithm dispatches the load economically, realizes the proportional active and reactive power sharing control, and frequency and voltage regulation, simultaneously.
3. The proposed algorithm embodies both self and communication time-delays and offers plug-and-play capability and robustness against communication link failure.
4. The proposed distributed control strategy exhibits a superior performance when compared with the distributed PI-based conventional control strategy.

### 1.3 Organization of the article

The remainder of the article is organized as follows: Section 2 describes a simplified dynamic BESS state-space model. Section 3 covers consensus based distributed control strategy for BESSs. Section 4 provides the stability analysis for different algorithms proposed in Section 3. Section 5 consists of different simulation results and discussion for validation of the proposed algorithms, and finally Section 6 provides the concluding remarks to this research work.

## 2 Dynamic state-space model of the BESS

A simplified dynamic state-space model of the *i*^*th*^ BESS, used in this research work, and proposed in [15], in compact vector-matrix form, can be expressed as follow:

$$\begin{array}{l} \underset{\dot{\text{x}}}{\underset{︸}{\left[\begin{array}{c} {\dot{E}}_{i}\\ {\dot{P}}_{i}\\ {\dot{Q}}_{i}\\ {\dot{\omega }}_{i}^{nom}\\ \left|{\dot{V}}_{i}^{nom}\right| \end{array}\right]}}=\underset{A}{\underset{︸}{\left[\begin{array}{ccccc} 0 & -\frac{1}{3600} & 0 & 0 & 0\\ 0 & 0 & 0 & 0 & 0\\ 0 & 0 & 0 & 0 & 0\\ 0 & 0 & 0 & 0 & 0\\ 0 & 0 & 0 & 0 & 0 \end{array}\right]}}\underset{\text{x}}{\underset{︸}{\left[\begin{array}{c} E_{i}\\ P_{i}\\ Q_{i}\\ \omega_{i}^{nom}\\ \left|V_{i}^{nom}\right| \end{array}\right]}}+\underset{B}{\underset{︸}{\left[\begin{array}{ccccc} 1 & 0 & 0 & 0 & 0\\ 0 & 1 & 0 & 0 & 0\\ 0 & 0 & 1 & 0 & 0\\ 0 & 0 & 0 & 1 & 0\\ 0 & 0 & 0 & 0 & 1 \end{array}\right]}}B\underset{\text{u}}{\underset{︸}{\left[\begin{array}{c} u_{i}^{E}\\ u_{i}^{P}\\ u_{i}^{Q}\\ u_{i}^{\omega }\\ u_{i}^{V} \end{array}\right]}}\\ \underset{\text{y}}{\underset{︸}{\left[\begin{array}{c} E_{i}\\ P_{i}\\ Q_{i}\\ \omega_{i}^{nom}\\ \left|V_{i}^{nom}\right| \end{array}\right]}}=\underset{\mathbb{C}}{\underset{︸}{\left[\begin{array}{ccccc} 1 & 0 & 0 & 0 & 0\\ 0 & 1 & 0 & 0 & 0\\ 0 & 0 & 1 & 0 & 0\\ 0 & 0 & 0 & 1 & 0\\ 0 & 0 & 0 & 0 & 1 \end{array}\right]}}\underset{\text{x}}{\underset{︸}{\left[\begin{array}{c} E_{i}\\ P_{i}\\ Q_{i}\\ \omega_{i}^{nom}\\ \left|V_{i}^{nom}\right| \end{array}\right]}} \end{array}\}$$ (1)

In Eq (1), *i* = {1, 2, …, *N*} is the index set of BESSs, $u_{i}^{E}$, $u_{i}^{P}$, $u_{i}^{Q}$, $u_{i}^{\omega }$, and $u_{i}^{V}$ are the consensus control inputs for energy level (SoC), active power, reactive power, frequency and voltage (i.e. *E*, *P*, *Q*, *ω* and *V*), respectively, designed in Section 3, and A, B, and C are constant matrices with compatible dimensions, and x∈R, u∈R and y∈R are, respectively, the state, the input and the output.

### 2.1 Droop control

When BESSs are operated in parallel, the droop control mechanism, which is implemented at the primary control level, serves as in charge of both the frequency and voltage regulation. In this mechanism, the frequency and voltage of each BESS is regulated by using the locally measured instantaneous (actual) active and reactive powers, respectively. The droop control mechanism basically mimics the operation of the governor and field exciter of a conventional synchronous generator to regulate the output frequency and voltage magnitude of a micro-generation source, according to the active and reactive power demands at its output terminals, respectively [20].

The frequency and voltage droop control dynamics for an *i*^*th*^ BESS can be expressed as follows:

$$\begin{array}{c} \omega_{i}=\underset{P-\omega \;\text{droop}\;\text{control}}{\underset{︸}{\omega_{i}^{nom}-K_{i}^{P}P_{i}}} \end{array}$$ (2)

$$\begin{array}{c} \left|V_{i}\right|=\underset{Q-V\;\text{droop}\;\text{control}}{\underset{︸}{\left|V_{i}^{nom}\right|-K_{i}^{Q}Q_{i}}} \end{array}$$ (3)

In Eqs (2) and (3), *ω*_*i*_ and *ω*^*nom*^ are the actual and nominal frequencies, |*V*_*i*_| and $\left|V_{i}^{nom}\right|$ are the actual and nominal voltage magnitudes, and $K_{i}^{P}$, $K_{i}^{Q}$ are the active and reactive power droop gains, and *P*_*i*_, *Q*_*i*_ represent the measured active and reactive powers, respectively, for an *i*^*th*^ BESS.

Note that the primary control provides the active and reactive power sharing, among all the BESSs, according to the following criteria:

$$\begin{array}{c} \left\{ \begin{array}{c} P_{i,sharing}=P_{j,sharing}\;or\;K_{i}^{P}P_{i}=K_{j}^{P}P_{j},\;\forall \;i=\left\{1,2,\ldots ,N\right\},\;j\in N_{i}\\ Q_{i,sharing}=Q_{j,sharing}\;or\;K_{i}^{Q}Q_{i}=K_{j}^{Q}Q_{j},\;\forall \;i=\left\{1,2,\ldots ,N\right\},\;j\in N_{i} \end{array}\right. \end{array}$$

where $j\in N_{i}$ represents the neighboring nodes set of node *i*. For ease of understanding, denoting the active and reactive power sharing for an *i*^*th*^ BESS by ${\tilde{P}}_{i}=K_{i}^{P}P_{i}$ and ${\tilde{Q}}_{i}=K_{i}^{Q}Q_{i}$, respectively.

In [17], it was assumed that the frequency and voltage deviation was always zero. Moreover, the dynamics of the droop controllers were ignored in the BESS model. However, the BESS model in this article explicitly considers and, hence, integrates the dynamics of the droop controllers into the simplified BESS model expressed in Eq (1). As a result, the final dynamic model of the droop-controlled *i*^*th*^ BESS, becomes as follows:

$$\begin{array}{c} \text{Dynamic}\;\text{model}\;\text{of}\;\text{the}\;\text{droop-controlled}\;i^{th}\;\text{BESS}\{\begin{array}{c} {\dot{E}}_{i}=-\frac{P_{i}}{3600}+u_{i}^{E}\\ {\dot{P}}_{i}=u_{i}^{P}\\ {\dot{Q}}_{i}=u_{i}^{Q}\\ {\dot{\omega }}_{i}^{nom}=u_{i}^{\omega }\\ \omega_{i}=\omega_{i}^{nom}-K_{i}^{P}P_{i}\\ \left|{\dot{V}}_{i}^{nom}\right|=u_{i}^{V}\\ \left|V_{i}\right|=\left|V_{i}^{nom}\right|-K_{i}^{Q}Q_{i} \end{array} \end{array}$$ (4)

It is also important to note that the existing LQR-based consensus control approaches in [17, 18] use only one control input $u_{i}^{P}$ for controlling both the energy and active power levels of the *i*^*th*^ BESS. In other words, these approaches control the energy levels of the BESSs indirectly through their powers. This strategy leads to a very slow convergence speed. Moreover, the design is not fully distributed. Hence, to increase the convergence speed, and to make the design fully distributed, the energy and active power level of the *i*^*th*^ BESS is controlled independently in this work by using two independent control inputs for each purpose (i.e. $u_{i}^{E}$, $u_{i}^{P}$). This is essential when the load demand varies rapidly and the DGs have to respond accordingly.

## 3 Consensus based distributed control strategy for BESSs

The consensus control inputs ($u_{i}^{E}$, $u_{i}^{P}$, $u_{i}^{Q}$, $u_{i}^{\omega }$, $u_{i}^{V}$) for BESSs are designed using the theory of multi-agent system (MAS), where each BESS is regarded as an agent, and the communication lines as edges. The communication/information exchange among various agents is covered using graph theory.

### 3.1 Preliminaries on graph theory

The information exchange among various agents (BESSs in this case) in a MAS, comprising *N* agents, can be graphically represented by a communication graph, and defined by algebraic graph theory. Such that, this graph is mathematically represented by a Laplacian matrix (or conductance matrix), *L* = [*ℓ*_*ij*_], with its diagonal and off-diagonal elements, defined below [21]:

$$\begin{array}{c} L=\left[\ell_{ij}\right]\{\begin{array}{c} \left(\text{diagonal}\;\text{elements})\;\ell_{ii}=d_{i}^{in}=\sum_{j\in N_{i}}a_{ij}\;\forall \;(i=j\right)\\ (\text{off-diagonal}\;\text{elements})\;\ell_{ij}=\{\begin{array}{cc} -a_{ij} & \forall \;i\neq j\;(\text{when}\;i\;\text{and}\;j\;\text{are}\\ & \text{connected})\\ 0 & \forall \;i\neq j\;(\text{when}\;i\;\text{and}\;j\;\text{are}\;\text{not}\\ & \text{connected}) \end{array} \end{array} \end{array}$$ (5)

In Eq (5), $d_{i}^{in}$ denotes the in-degree of a node *i* (i.e. the number of neighboring nodes, it is receiving information from) and *a*_*ij*_ stands for the weight of the communication link between two nodes *i* and *j*. An equivalent method for constructing the Laplacian matrix is using, *L* = *D* − *A*, where $D=diag\{d_{i}^{in}\}$ and *A* = [*a*_*ij*_] stand for the in-degree matrix and adjacency (or connectivity) matrix, respectively. The adjacency matrix, with its diagonal and off-diagonal elements, can be defined as follows [22]:

$$\begin{array}{c} A=\left[a_{ij}\right]\{\begin{array}{c} (\text{diagonal}\;\text{elements})\;a_{ii}=0\\ (\text{off-diagonal}\;\text{elements})\;a_{ij}=\{\begin{array}{cc} >0 & \forall \;i\neq j\;(\text{when}\;i\;\text{and}\;j\;\text{are}\\ & \text{connected})\\ 0 & \forall \;i\neq j\;(\text{when}\;i\;\text{and}\;j\;\text{are}\;\text{not}\\ & \text{connected}) \end{array} \end{array} \end{array}$$ (6)

The in-degree matrix is a diagonal matrix where the diagonal elements denote the in-degree, $d_{i}^{in}$, of each node. On the other hand, the adjacency matrix is a non-negative matrix with all the diagonal elements equal to zero. The out-degree of a node *i*, represented by $d_{i}^{out}$, denotes the number of neighboring nodes, it is sending information to. If the in-degree and out-degree are equal, for all nodes, the communication graph is known as balanced. A communication graph is termed as undirected, if *a*_*ij*_ = *a*_*ji*_, that is if there is a bidirectional/two-way communication link between nodes *i* and *j* having the same weights in both directions. Otherwise, for *a*_*ij*_ ≠ *a*_*ji*_ the communication graph is termed as a directed graph or a digraph. A directed graph is known as strongly connected, if there exists a directed path between every two distinct nodes. Similarly, an undirected graph is known as simply connected, if there exists an undirected path between every two distinct nodes (i.e. a directed path from node *i* to *j*, and also a directed path from node *j* to *i*). The in-degree of a node *i* equals the *i*^*th*^ row sum of the adjacency matrix, whereas, its out-degree equals the *i*^*th*^ column sum of the adjacency matrix. For a balanced undirected graph, the adjacency matrix is symmetric (i.e. *A* = *A*^*T*^), moreover, its *i*^*th*^ row sum equals the *i*^*th*^ column sum.

In general, for any Laplacian matrix whether balanced or not, all row sums are equal to zero. Furthermore, for a balanced Laplacian matrix, all the row sums as well as all the column sums are equal to zero. A balanced undirected graph has a symmetric positive semi-definite Laplacian matrix. If the communication graph is connected, it possesses a simple 0 eigenvalue with a corresponding eigenvector **1** (where **1** represents a column vector of ones), while all the other eigenvalues are positive.

The Laplacian matrix of a connected and balanced communication graph satisfies the following condition [17]:

$$\begin{array}{c} L1=0 \end{array}$$ (7)

and

$$\begin{array}{c} 1^{T}L={\left(L1\right)}^{T}=0 \end{array}$$ (8)

### 3.2 Time-delayed consensus design

The consensus development is based on the communication among various BESSs. Therefore, the communication time-delays may potentially deteriorate the convergence and performance of the consensus algorithms, especially when the time-delays are very large. To ensure the smart grid reliability and avoid any potential instability issue, the Institution of Electrical and Electronics Engineers (IEEE) and International Electro-technical Commission (IEC) have clearly defined standards for communication delay requirements. These two well-known standards set the maximum permissible communication delay to be 16 milliseconds for both monitoring and control purposes [23]. The same standard has also been taken into consideration in this article.

Two different types of time-delays (i.e self time-delay, *T*_*ij*_ > 0, and communication time-delay, *τ*_*ij*_ > 0) have been explicitly considered in the proposed work. These time-delays are imposed on the states of a node or agent *i* (BESS in this case) and the neighboring node $j\in N_{i}$. The self time-delay is the delay occurring on the computation or reaction of a node. On the other hand, the communication time-delay is the delay on the communication channel when a state propagates from a node *j* to a neighboring node *i*.

In the existing literature, a time-delayed robust consensus design for SoC balancing of ESSs in a microgrid can be found in [24]. However, this approach considers only the communication time delays (*τ*_*ij*_ > 0), and not the self time delays (*T*_*ij*_ = 0).

### 3.3 Leader-follower based consensus design

Generally, the droop control (primary control) alone may not restore the frequencies and voltage magnitudes of the BESSs back to their nominal values, especially when there is a load change. Hence, a secondary control level is always required, in addition to primary control, for frequencies and voltage magnitudes restoration. In the proposed design, the secondary frequency and voltage support/restoration is accomplished by using a leader-follower consensus approach.

In the leader-follower consensus approach, the frequencies and voltage magnitudes of all the BESSs are regulated by selecting a virtual leader node (BESS) in the system of multiple BESSs. This leader node must be connected to at least one other node. The leader node provides/dictates a reference frequency and voltage magnitude (i.e. *ω*_*ref*_ and |*V*_*ref*_|, respectively) to the distributed controller. The distributed controller also receives information from all the neighboring nodes. In this way, the frequencies and voltage magnitudes of all the remaining BESSs (followers) are then tracked through the distributed controller to these references commanded by the leader node, even if there is a load change, provided that the communication graph among the BESSs is connected.

The existence of the leader node is represented by an index *α*_0*i*_, such that, *α*_0*i*_ = 1 if a node *i* is connected to the leader (i.e. $i\in N_{0}$), otherwise *α*_0*i*_ = 0 (i.e. $i\notin N_{0}$), where $N_{0}$ represents the neighboring set of nodes of the leader node.

### 3.4 Consensus control inputs design

The dynamic closed-loop model of the *i*^*th*^ BESS with distributed control feature, and based on time-delayed leader-follower consensus approach, for fulfilling different objectives, is formulated by designing the consensus control inputs given in Eq (4) ($u_{i}^{E}$, $u_{i}^{P}$, $u_{i}^{Q}$, $u_{i}^{\omega }$, $u_{i}^{V}$) as follows:

$$\begin{array}{c} \begin{array}{cc} & {\dot{E}}_{i}\left(t\right)=-\frac{P_{i}\left(t\right)}{3600}+u_{i}^{E}\left(t\right)=-\frac{P_{i}\left(t\right)}{3600}-\frac{C_{1}}{K_{i}^{P}}\sum_{j\in N_{i}}\frac{1}{d_{i}^{in}}[K_{i}^{P}E_{i}\left(t-T_{ij}\right)-K_{j}^{P}E_{j}\left(t-\tau_{ij}\right)]\\ & -C_{0i}^{E}[\underset{\text{Actual}\;\text{SoC}}{\underset{︸}{E_{i}\left(t-T_{ij}\right)}}-\underset{\text{Reference}\;\text{SoC}}{\underset{︸}{E_{i_{ref}}}}] \end{array} \end{array}$$ (9)

$$\begin{array}{c} \begin{array}{cc} & {\dot{P}}_{i}\left(t\right)=u_{i}^{P}\left(t\right)=-\frac{C_{2}}{K_{i}^{P}}\sum_{j\in N_{i}}\frac{1}{d_{i}^{in}}[K_{i}^{P}P_{i}\left(t-T_{ij}\right)-K_{j}^{P}P_{j}\left(t-\tau_{ij}\right)] \end{array} \end{array}$$ (10)

$$\begin{array}{c} \begin{array}{cc} & {\dot{Q}}_{i}\left(t\right)=u_{i}^{Q}\left(t\right)=-\frac{C_{3}}{K_{i}^{Q}}\sum_{j\in N_{i}}\frac{1}{d_{i}^{in}}[K_{i}^{Q}Q_{i}\left(t-T_{ij}\right)-K_{j}^{Q}Q_{j}\left(t-\tau_{ij}\right)] \end{array} \end{array}$$ (11)

$$\begin{array}{c} \begin{array}{cc} & {\dot{\omega }}_{i}^{nom}\left(t\right)=u_{i}^{\omega }\left(t\right)=-C_{2}\sum_{j\in N_{i}}\frac{1}{d_{i}^{in}}[\omega_{i}^{nom}\left(t-T_{ij}\right)-\omega_{j}^{nom}\left(t-\tau_{ij}\right)]\\ & -C_{0}^{\omega }\alpha_{0i}[\omega_{i}\left(t-T_{ij}\right)-\omega_{ref}]-u_{i}^{Cp}\left(t\right) \end{array} \end{array}$$ (12)

In Eq (12), *ω*_*i*_(*t* − *T*_*ij*_) is given as follows:

$$\begin{array}{c} \omega_{i}\left(t-T_{ij}\right)=\underset{P-\omega \;\text{droop}\;\text{control}}{\underset{︸}{\omega_{i}^{nom}\left(t-T_{ij}\right)-K_{i}^{P}P_{i}\left(t-T_{ij}\right)}} \end{array}$$ (13)

$$\begin{array}{c} \begin{array}{cc} & \left|{\dot{V}}_{i}^{nom}\left(t\right)\right|=u_{i}^{V}\left(t\right)=-C_{3}\sum_{j\in N_{i}}\frac{1}{d_{i}^{in}}[\left|V_{i}^{nom}\left(t-T_{ij}\right)\right|-\left|V_{j}^{nom}\left(t-\tau_{ij}\right)\right|]\\ & -C_{0}^{V}\alpha_{0i}[\left|V_{i}\left(t-T_{ij}\right)\right|-\left|V_{ref}\right|] \end{array} \end{array}$$ (14)

In Eq (14), |*V*_*i*_|(*t* − *T*_*ij*_) is expressed as follows:

$$\begin{array}{c} \left|V_{i}\left(t-T_{ij}\right)\right|=\underset{Q-V\;\text{droop}\;\text{control}}{\underset{︸}{\left|V_{i}^{nom}\left(t-T_{ij}\right)\right|-K_{i}^{Q}Q_{i}\left(t-T_{ij}\right)}} \end{array}$$ (15)

Eq (9) is the SoC trajectory tracking control algorithm, whereas Eqs (10), (11), (12) and (14) are the consensus control algorithms for active power, reactive power, frequency and voltage magnitude regulation, respectively, where *E*_*i*_, *P*_*i*_ and *Q*_*i*_ represent the actual (or measured) energy level (or SoC), actual active power and actual reactive power of the *i*^*th*^ BESS, respectively. Assuming that the BESS energy base equals its capacity, then the BESS (per unit) energy can be taken equal to its SoC (i.e. *E*_*i*_ = *SoC*_*i*_), as reported in [17]. The actual frequency and voltage magnitude at the *i*^*th*^ BESS terminals are denoted by *ω*_*i*_ and |*V*_*i*_|, respectively. The nominal frequency and voltage magnitude of the *i*^*th*^ BESS are designated by $\omega_{i}^{nom}$ and $\left|V_{i}^{nom}\right|$, respectively. While $C_{1}$, $C_{2}$, and $C_{3}>0$ correspond to positive scalar coupling/consensus gains.

It should be noted that the consensus algorithms for active power, reactive power and voltage, respectively, expressed in Eqs (10), (11) and (14) are exactly the same as proposed in [15]. However, the algorithms given in Eqs (9) and (12) for energy level (i.e. SoC) and frequency, respectively, have been modified. The reason behind this modification is to achieve the objectives of SoC trajectory tracking control and the ELD for the BESSs. For achieving the objective of the ELD, an additional consensus control input, $u_{i}^{Cp}\left(t\right)$, that also appears in Eq (12), is considered in this research article. It is defined in Eq (16), as follows:

$$\begin{array}{c} \begin{array}{cc} & u_{i}^{Cp}\left(t\right)=-C_{4}\sum_{j\in N_{i}}\frac{1}{d_{i}^{in}}[\lambda_{i}P_{i,sharing}\left(t-T_{ij}\right)-\lambda_{j}P_{j,sharing}\left(t-\tau_{ij}\right)]\\ & =-C_{4}\sum_{j\in N_{i}}\frac{1}{d_{i}^{in}}[C_{p_{i}}\left(t-T_{ij}\right)-C_{p_{j}}\left(t-\tau_{ij}\right)] \end{array} \end{array}$$ (16)

In Eq (16), $u_{i}^{Cp}$ is the production cost consensus control input, $C_{4}>0$ is a positive scalar coupling/consensus gain, $C_{p_{i}}$ and λ_*i*_ are the production cost and incremental cost of the *i*^*th*^ BESS, respectively, and $P_{i,sharing}=K_{i}^{P}P_{i}$ is the power shared by the *i*^*th*^ BESS.

**Fig 1:**
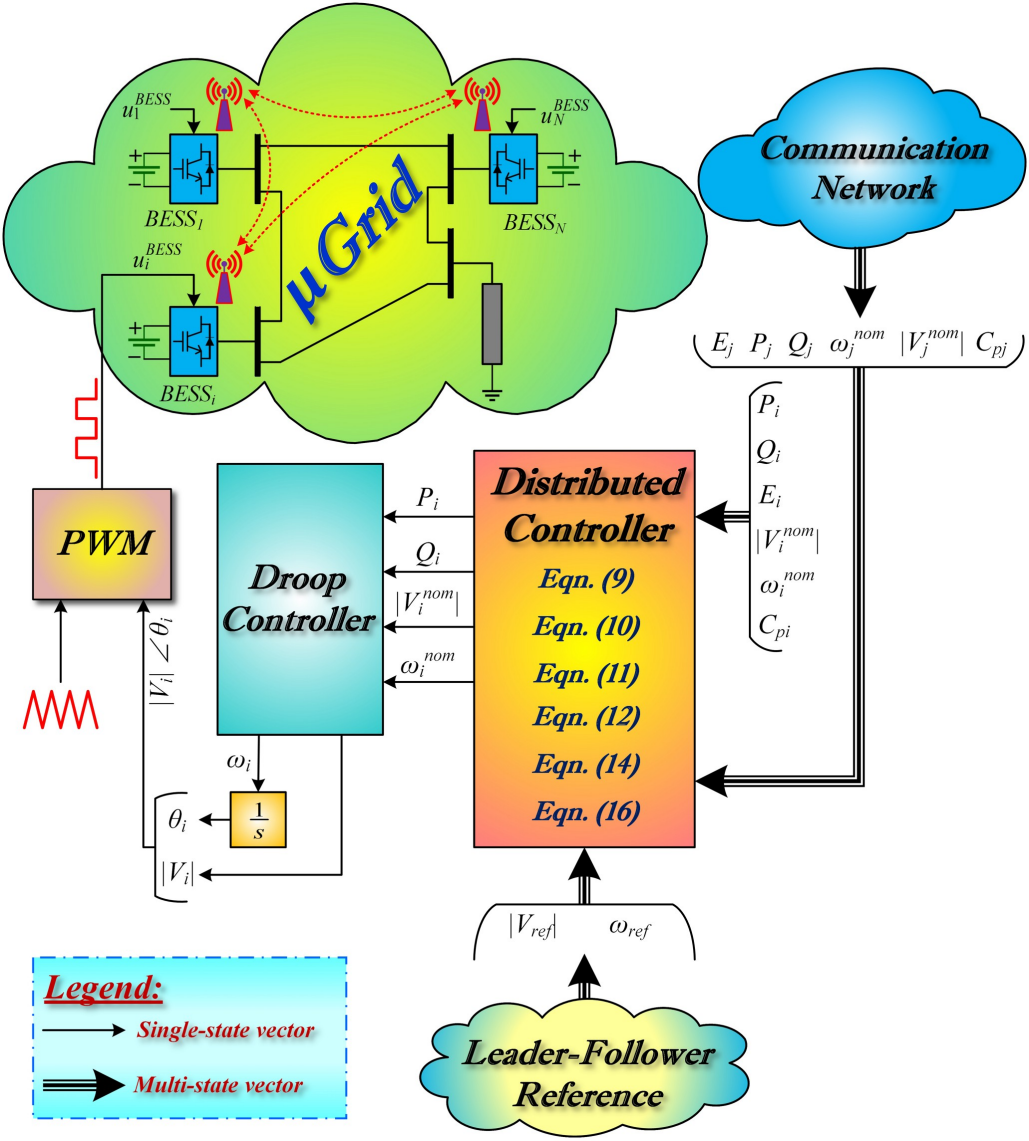
Closed-loop BESS model with leader-follower consensus approach.

The detailed closed-loop BESS model with distributed control feature, and based on leader-follower consensus approach, is depicted in Fig 1. Note that the consensus algorithms, Eqs (9)–(12) and (14), work on the basis of integral control. This integral control is assumed to be installed at each battery converter. It receives both local information (from *i*^*th*^ BESSs) and the neighbors information (from *j*^*th*^ BESSs), to fulfill the following control objectives:

1. Frequencies and voltage magnitudes of all the BESSs must track their desired reference values, that is, (*ω*_1_ = *ω*_2_ = , …, = *ω*_*N*_ = *ω*_*ref*_) and (|*V*_1_| = |*V*_2_| = , …, = |*V*_*N*_| = |*V*_*ref*_|)
2. SoCs of all the BESSs must track their reference trajectories, that is, ${SoC}_{i}={SoC}_{i_{ref}}$
3. Equal active and reactive power sharing among BESSs must be guaranteed, that is, $\left(K_{1}^{P}P_{1}=K_{2}^{P}P_{2}=,\ldots ,=K_{N}^{P}P_{N}\right)$ and $\left(K_{1}^{Q}Q_{1}=K_{2}^{Q}Q_{2}=,\ldots ,=K_{N}^{Q}Q_{N}\right)$
4. Load must be economically dispatched, that is, (*Cp*_1_ = *Cp*_2_ = , …, = *Cp*_*N*_)

## 4 Stability analysis

In this section, stability analysis of different algorithms proposed in Section 3.4 is presented. It is evident from Eqs (10), (11) and (16) that the active power, reactive power and the production cost consensus control inputs of the *i*^*th*^ BESS have been expressed in the same form. Hence, stability proof for active power sharing will be provided only, while for reactive power sharing and production cost, it can be derived in the same manner.

To transform these algorithms from the time-domain into the Laplace domain, the delay-dependent adjacency, the in-degree and the Laplacian matrices of the communication graph, discussed in Section 3.1, are, respectively, expressed as follows:

$$\begin{array}{c} A_{d}\left(s\right)=[e^{-s\tau_{ij}}] \end{array}$$ (17)

$$\begin{array}{c} D_{d}\left(s\right)=diag\{\sum_{j\in N_{i}}e^{-sT_{ij}}\} \end{array}$$ (18)

$$\begin{array}{c} L_{d}\left(s\right)=D_{d}\left(s\right)-A_{d}\left(s\right) \end{array}$$ (19)

### 4.1 Stability proof for SoC trajectory tracking control of the BESSs

Substituting $K_{i}^{P}E_{i}={\tilde{E}}_{i}$, $K_{i}^{P}P_{i}={\tilde{P}}_{i}$, $K_{j}^{P}E_{j}={\tilde{E}}_{j}$ and $K_{i}^{P}E_{i_{ref}}={\tilde{E}}_{i_{ref}}$, Eq (9) can be re-written as follows:

$$\begin{array}{c} {\dot{\tilde{E}}}_{i}\left(t\right)=-\frac{{\tilde{P}}_{i}\left(t\right)}{3600}-C_{1}\sum_{j\in N_{i}}\frac{1}{d_{i}^{in}}[{\tilde{E}}_{i}\left(t-T_{ij}\right)-{\tilde{E}}_{j}\left(t-\tau_{ij}\right)]-C_{0i}^{E}[{\tilde{E}}_{i}\left(t-T_{ij}\right)-{\tilde{E}}_{i_{ref}}] \end{array}$$ (20)

Eq (20) represents a closed-loop equation for SoC trajectory tracking control of the BESSs. If $\tilde{E}=[K_{1}^{P}E_{1},K_{2}^{P}E_{2},\ldots ,K_{N}^{P}E_{N}{]}^{T}$, then Eq (20), in time-domain, takes the following generalized form:

$$\begin{array}{c} \dot{\tilde{E}}\left(t\right)=-\frac{\tilde{P}\left(t\right)}{3600}-C_{1}D^{-1}L_{d}\left(t\right)\tilde{E}\left(t\right)-C_{0}^{E}D_{d}\left(t\right)\tilde{E}\left(t\right)+C_{0}^{E}{\tilde{E}}_{ref}\left(t\right) \end{array}$$ (21)

Re-writing Eq (21) in Laplace domain as follows:

$$\begin{array}{c} s\tilde{E}\left(s\right)=-\frac{\tilde{P}\left(s\right)}{3600}-C_{1}D^{-1}L_{d}\left(s\right)\tilde{E}\left(s\right)-C_{0}^{E}D_{d}\left(s\right)\tilde{E}\left(s\right)+C_{0}^{E}{\tilde{E}}_{ref}\left(s\right) \end{array}$$ (22)

Eq (22) can also be expressed as follows:

$$\begin{array}{c} \tilde{E}\left(s\right)[sI+C_{1}D^{-1}L_{d}\left(s\right)+C_{0}^{E}D_{d}\left(s\right)]=(-\frac{\tilde{P}\left(s\right)}{3600}+C_{0}^{E}{\tilde{E}}_{ref}\left(s\right)) \end{array}$$ (23)

Rearranging and simplifying Eq (23), it can be expressed as follows:

$$\begin{array}{c} \tilde{E}\left(s\right)=[sI+C_{1}D^{-1}L_{d}\left(s\right)+C_{0}^{E}D_{d}\left(s\right){]}^{-1}(-\frac{\tilde{P}\left(s\right)}{3600}+C_{0}^{E}{\tilde{E}}_{ref}) \end{array}$$ (24)

where $C_{1},\;C_{0}^{E}>0$. Hence, SoC trajectory tracking control is achieved only, if the communication graph is connected. Moreover, the characteristic equation of the closed-loop system, that is, $\Delta \left(s\right)=det(sI+C_{1}D^{-1}L_{d}\left(s\right)+C_{0}^{E}D_{d}\left(s\right))=0$ has no roots in the right-half of the complex plane.

### 4.2 Consensus proof for active power sharing control of the BESSs

Substituting $K_{i}^{P}P_{i}={\tilde{P}}_{i}$ and $K_{j}^{P}P_{j}={\tilde{P}}_{j}$, Eq (10) can be re-written as follows:

$$\begin{array}{c} {\dot{\tilde{P}}}_{i}\left(t\right)=-C_{2}\sum_{j\in N_{i}}\frac{1}{d_{i}^{in}}[{\tilde{P}}_{i}\left(t-T_{ij}\right)-{\tilde{P}}_{j}\left(t-\tau_{ij}\right)] \end{array}$$ (25)

Eq (25) represents a closed-loop equation for active power sharing control of the BESSs. If $\tilde{P}=[K_{1}^{P}P_{1},K_{2}^{P}P_{2},\ldots ,K_{N}^{P}P_{N}{]}^{T}$, then Eq (25), in time-domain, takes the following generalized form:

$$\begin{array}{c} \dot{\tilde{P}}\left(t\right)=-C_{2}D^{-1}L_{d}\left(t\right)\tilde{P}\left(t\right) \end{array}$$ (26)

Re-writing Eq (26) in Laplace domain as follows:

$$\begin{array}{c} s\tilde{P}\left(s\right)=-C_{2}D^{-1}L_{d}\left(s\right)\tilde{P}\left(s\right) \end{array}$$ (27)

Rearranging and simplifying Eq (27), it can be expressed as follows:

$$\begin{array}{c} \tilde{P}\left(s\right)(sI+C_{2}D^{-1}L_{d}\left(s\right))=0 \end{array}$$ (28)

where $C_{2}>0$. Therefore, the active power sharing consensus is achieved only, if the communication graph is connected. Furthermore, the characteristic equation of the closed-loop BESSs, that is, $\Delta \left(s\right)=det(sI+C_{2}D^{-1}L_{d}\left(s\right))=0$ has no roots in the right-half of the s-plane.

### 4.3 Consensus proof for frequency tracking/regulation of the BESSs

Taking time derivative of Eq (13), it can be re-written as follows:

$$\begin{array}{c} {\dot{\omega }}_{i}\left(t\right)={\dot{\omega }}_{i}^{nom}\left(t\right)-{\dot{\tilde{P}}}_{i}\left(t\right) \end{array}$$ (29)

Also, let

$$\begin{array}{c} {\hat{\omega }}_{i}\left(t\right)=\omega_{i}\left(t\right)-\omega_{ref}\left(t\right) \end{array}$$ (30)

Rearranging and taking time derivative of Eq (30), it yields:

$$\begin{array}{c} {\dot{\omega }}_{i}\left(t\right)={\dot{\hat{\omega }}}_{i}\left(t\right)+{\dot{\omega }}_{ref}\left(t\right) \end{array}$$ (31)

Now substituting the values of ${\dot{\omega }}_{i}\left(t\right)$ from Eq (31), ${\dot{\omega }}_{i}^{nom}\left(t\right)$ from Eq (12), $u_{i}^{Cp}\left(t\right)$ from Eq (16) and ${\dot{\tilde{P}}}_{i}\left(t\right)$ from Eq (25) into Eq (29), the following equation is obtained:

$$\begin{array}{c} \begin{array}{cc} & {\dot{\hat{\omega }}}_{i}\left(t\right)+{\dot{\omega }}_{ref}\left(t\right)=-C_{2}\sum_{j\in N_{i}}\frac{1}{d_{i}^{in}}[\omega_{i}^{nom}\left(t-T_{ij}\right)-\omega_{j}^{nom}\left(t-\tau_{ij}\right)]-C_{0}^{\omega }\alpha_{0i}{\hat{\omega }}_{i}\left(t-T_{ij}\right)-\\ & -C_{4}\sum_{j\in N_{i}}\frac{1}{d_{i}^{in}}[\lambda_{i}P_{i}\left(t-T_{ij}\right)-\lambda_{j}P_{j}\left(t-\tau_{ij}\right)]-C_{2}\sum_{j\in N_{i}}\frac{1}{d_{i}^{in}}[{\tilde{P}}_{i}\left(t-T_{ij}\right)-{\tilde{P}}_{j}\left(t-\tau_{ij}\right)] \end{array} \end{array}$$ (32)

Simplifying Eq (32) using $\omega_{i}^{nom}\left(t-T_{ij}\right)-{\tilde{P}}_{i}\left(t-T_{ij}\right)=\omega_{i}\left(t-T_{ij}\right)$ and $\omega_{j}^{nom}\left(t-\tau_{ij}\right)-{\tilde{P}}_{j}\left(t-\tau_{ij}\right)=\omega_{j}\left(t-\tau_{ij}\right)$ from Eq (13), $\lambda_{i}P_{i}=P_{i}^{*}$ and $\lambda_{j}P_{j}=P_{j}^{*}$, it can be expressed as follows:

$$\begin{array}{c} \begin{array}{cc} & {\dot{\hat{\omega }}}_{i}\left(t\right)+{\dot{\omega }}_{ref}\left(t\right)=-C_{2}\sum_{j\in N_{i}}\frac{1}{d_{i}^{in}}[\omega_{i}\left(t-T_{ij}\right)-\omega_{j}\left(t-\tau_{ij}\right)]-C_{2}\sum_{j\in N_{i}}\frac{1}{d_{i}^{in}}[\omega_{ref}\left(t-T_{ij}\right)\\ & -\omega_{ref}\left(t-T_{ij}\right)+\omega_{ref}\left(t-\tau_{ij}\right)-\omega_{ref}\left(t-\tau_{ij}\right)]-C_{0}^{\omega }\alpha_{0i}{\hat{\omega }}_{i}\left(t-T_{ij}\right)\\ & -C_{4}\sum_{j\in N_{i}}\frac{1}{d_{i}^{in}}[P_{i}^{*}\left(t-T_{ij}\right)-P_{j}^{*}\left(t-\tau_{ij}\right)] \end{array} \end{array}$$ (33)

Using $\omega_{i}\left(t-T_{ij}\right)-\omega_{ref}\left(t-T_{ij}\right)={\hat{\omega }}_{i}\left(t-T_{ij}\right)$ and $\omega_{j}\left(t-\tau_{ij}\right)-\omega_{ref}\left(t-\tau_{ij}\right)={\hat{\omega }}_{j}\left(t-\tau_{ij}\right)$, Eq (33) can be further simplified as follows:

$$\begin{array}{c} \begin{array}{cc} & {\dot{\hat{\omega }}}_{i}\left(t\right)+{\dot{\omega }}_{ref}\left(t\right)=-C_{2}\sum_{j\in N_{i}}\frac{1}{d_{i}^{in}}[{\hat{\omega }}_{i}\left(t-T_{ij}\right)-{\hat{\omega }}_{j}\left(t-\tau_{ij}\right)]-C_{2}\sum_{j\in N_{i}}\frac{1}{d_{i}^{in}}[\omega_{ref}\left(t-T_{ij}\right)\\ & -\omega_{ref}\left(t-\tau_{ij}\right)]-C_{0}^{\omega }\alpha_{0i}{\hat{\omega }}_{i}\left(t-T_{ij}\right)-C_{4}\sum_{j\in N_{i}}\frac{1}{d_{i}^{in}}[P_{i}^{*}\left(t-T_{ij}\right)-P_{j}^{*}\left(t-\tau_{ij}\right)] \end{array} \end{array}$$ (34)

If $\hat{\omega }=[{\hat{\omega }}_{1},{\hat{\omega }}_{2},\ldots ,{\hat{\omega }}_{N}{]}^{T}$ and P* = [λ_1_ *P*_1_, λ_2_ *P*_2_ …, λ_*N*_ *P*_*N*_]^*T*^ then Eq (34), in time-domain, takes the following generalized form:

$$\begin{array}{c} \begin{array}{cc} & \dot{\hat{\omega }}\left(t\right)+{\dot{\omega }}_{ref}\left(t\right)=-C_{2}D^{-1}L_{d}\left(t\right)\hat{\omega }\left(t\right)-C_{2}D^{-1}L_{d}\left(t\right)\omega_{ref}\left(t\right)-C_{0}^{\omega }D_{0}D_{d}\left(t\right)\hat{\omega }\left(t\right)\\ & -C_{4}\left(t\right)D^{-1}L_{d}\left(t\right)P^{*}\left(t\right) \end{array} \end{array}$$ (35)

where *D*_0_ = *diag*{*α*_0*i*_}. Re-writing Eq (35) in Laplace domain as follows:

$$\begin{array}{c} \begin{array}{cc} & s\hat{\omega }\left(s\right)+s\omega_{ref}\left(s\right)=-C_{2}D^{-1}L_{d}\left(s\right)\hat{\omega }\left(s\right)-C_{2}D^{-1}L_{d}\left(s\right)\omega_{ref}\left(s\right)-C_{0}^{\omega }D_{0}D_{d}\left(s\right)\hat{\omega }\left(s\right)\\ & -C_{4}\left(s\right)D^{-1}L_{d}\left(s\right)P^{*}\left(s\right) \end{array} \end{array}$$ (36)

Rearranging and simplifying Eq (36), it reduces to the following form:

$$\begin{array}{c} \begin{array}{cc} & \hat{\omega }\left(s\right)=-[sI+C_{2}D^{-1}L_{d}\left(s\right)+C_{0}^{\omega }D_{0}D_{d}\left(s\right){]}^{-1}[(sI+C_{2}D^{-1}L_{d}\left(s\right))\omega_{ref}\left(s\right)\\ & +C_{4}\left(s\right)D^{-1}L_{d}\left(s\right)P^{*}\left(s\right)] \end{array} \end{array}$$ (37)

where $C_{2},\;C_{0}^{\omega },\;C_{4}>0$. Therefore, the frequency consensus is achieved only, if the communication graph is connected. Furthermore, the characteristic equation of the closed-loop system, that is, $\Delta \left(s\right)=det(sI+C_{2}D^{-1}L_{d}\left(s\right)+C_{0}^{\omega }D_{0}D_{d}\left(s\right))=0$ has no roots in the right-half of the s-plane.

### 4.4 Consensus proof for voltage tracking/regulation of the BESSs

Taking time derivative of Eq (15), it can be re-written as follows:

$$\begin{array}{c} \left|{\dot{V}}_{i}\left(t\right)\right|=\left|{\dot{V}}_{i}^{nom}\left(t\right)\right|-{\dot{\tilde{Q}}}_{i}\left(t\right) \end{array}$$ (38)

where,

$$\begin{array}{c} {\dot{\tilde{Q}}}_{i}\left(t\right)=-C_{3}\sum_{j\in N_{i}}\frac{1}{d_{i}^{in}}[{\tilde{Q}}_{i}\left(t-T_{ij}\right)-{\tilde{Q}}_{j}\left(t-\tau_{ij}\right)] \end{array}$$ (39)

Also, let

$$\begin{array}{c} \left|{\hat{V}}_{i}\left(t\right)\right|=\left|V_{i}\left(t\right)\right|-\left|V_{ref}\left(t\right)\right| \end{array}$$ (40)

Rearranging and taking time derivative of Eq (40), it yields:

$$\begin{array}{c} {\dot{V}}_{i}\left(t\right)={\dot{\hat{V}}}_{i}\left(t\right)+\left|{\dot{V}}_{ref}\left(t\right)\right| \end{array}$$ (41)

Now substituting the values of ${\dot{V}}_{i}\left(t\right)$ from Eq (41), ${\dot{V}}_{i}^{nom}\left(t\right)$ from Eq (14), and ${\dot{\tilde{Q}}}_{i}\left(t\right)$ from Eq (39) into Eq (38), it yields the following equation:

$$\begin{array}{c} \begin{array}{cc} & \left|{\dot{\hat{V}}}_{i}\left(t\right)\right|+\left|{\dot{V}}_{ref}\left(t\right)\right|=-C_{3}\sum_{j\in N_{i}}\frac{1}{d_{i}^{in}}[\left|V_{i}^{nom}\left(t-T_{ij}\right)\right|-\left|V_{j}^{nom}\left(t-\tau_{ij}\right)\right|]\\ & -C_{0}^{V}\alpha_{0i}{\hat{V}}_{i}\left(t-T_{ij}\right)-C_{3}\sum_{j\in N_{i}}\frac{1}{d_{i}^{in}}[{\tilde{Q}}_{i}\left(t-T_{ij}\right)-{\tilde{Q}}_{j}\left(t-\tau_{ij}\right)] \end{array} \end{array}$$ (42)

Simplifying Eq (42) using $\left|V_{i}^{nom}\left(t-T_{ij}\right)\right|-{\tilde{Q}}_{i}\left(t-T_{ij}\right)=\left|V_{i}\left(t-T_{ij}\right)\right|$ and $\left|V_{j}^{nom}\left(t-\tau_{ij}\right)\right|-{\tilde{Q}}_{j}\left(t-\tau_{ij}\right)=\left|V_{j}\left(t-\tau_{ij}\right)\right|$ from Eq (15), it can be expressed as follows:

$$\begin{array}{c} \begin{array}{cc} & \left|{\dot{\hat{V}}}_{i}\left(t\right)\right|+\left|{\dot{V}}_{ref}\left(t\right)\right|=-C_{3}\sum_{j\in N_{i}}\frac{1}{d_{i}^{in}}[\left|V_{i}\left(t-T_{ij}\right)\right|-\left|V_{j}\left(t-\tau_{ij}\right|\right)]\\ & -C_{3}\sum_{j\in N_{i}}\frac{1}{d_{i}^{in}}[\left|V_{ref}\left(t-T_{ij}\right)\right|-\left|V_{ref}\left(t-T_{ij}\right)\right|+\left|V_{ref}\left(t-\tau_{ij}\right)\right|-\left|V_{ref}\left(t-\tau_{ij}\right)\right|]\\ & -C_{0}^{V}\alpha_{0i}\left|{\hat{V}}_{i}\left(t-T_{ij}\right)\right| \end{array} \end{array}$$ (43)

Using $\left|V_{i}\left(t-T_{ij}\right)\right|-\left|V_{ref}\left(t-T_{ij}\right)\right|=\left|{\hat{V}}_{i}\left(t-T_{ij}\right)\right|$ and $\left|V_{j}\left(t-\tau_{ij}\right)\right|-\left|V_{ref}\left(t-\tau_{ij}\right)\right|=\left|{\hat{V}}_{j}\left(t-\tau_{ij}\right)\right|$, Eq (43) can be further simplified as follows:

$$\begin{array}{c} \begin{array}{cc} & \left|{\dot{\hat{V}}}_{i}\left(t\right)\right|+\left|{\dot{V}}_{ref}\left(t\right)\right|=-C_{3}\sum_{j\in N_{i}}\frac{1}{d_{i}^{in}}[\left|{\hat{V}}_{i}\left(t-T_{ij}\right)\right|-\left|{\hat{V}}_{j}\left(t-\tau_{ij}\right)\right|]\\ & -C_{3}\sum_{j\in N_{i}}\frac{1}{d_{i}^{in}}[\left|V_{ref}\left(t-T_{ij}\right)\right|-\left|V_{ref}\left(t-\tau_{ij}\right)\right|]-C_{0}^{V}\alpha_{0i}\left|{\hat{V}}_{i}\left(t-T_{ij}\right)\right| \end{array} \end{array}$$ (44)

If $\left|\hat{V}\right|=[\left|{\hat{V}}_{1}\right|,\left|{\hat{V}}_{2}\right|,\ldots ,\left|{\hat{V}}_{N}\right|{]}^{T}$, then Eq (44), in time-domain, takes the following generalized form:

$$\begin{array}{c} \begin{array}{cc} & \left|\dot{\hat{V}}\left(t\right)\right|+\left|{\dot{V}}_{ref}\left(t\right)\right|=-C_{3}D^{-1}L_{d}\left(t\right)\left|\hat{V}\left(t\right)\right|-C_{3}D^{-1}L_{d}\left(t\right)\left|V_{ref}\left(t\right)\right|-C_{0}^{V}D_{0}D_{d}\left(t\right)\left|\hat{V}\left(t\right)\right| \end{array} \end{array}$$ (45)

where *D*_0_ = *diag*{*α*_0*i*_}. Re-writing Eq (45) in Laplace domain as follows:

$$\begin{array}{c} \begin{array}{cc} & s\left|\hat{V}\left(s\right)\right|+s\left|V_{ref}\left(s\right)\right|=-C_{3}D^{-1}L_{d}\left(s\right)\left|\hat{V}\left(s\right)\right|-C_{3}D^{-1}L_{d}\left(s\right)\left|V_{ref}\left(s\right)\right|\\ & -C_{0}^{V}D_{0}D_{d}\left(s\right)\left|\hat{V}\left(s\right)\right| \end{array} \end{array}$$ (46)

Rearranging and simplifying Eq (46), it reduces to the following form:

$$\begin{array}{c} \begin{array}{cc} & \left|\hat{V}\left(s\right)\right|=-[sI+C_{3}D^{-1}L_{d}\left(s\right)+C_{0}^{V}D_{0}D_{d}\left(s\right){]}^{-1}[(sI+C_{3}D^{-1}L_{d}\left(s\right))\left|V_{ref}\left(s\right)\right|] \end{array} \end{array}$$ (47)

where $C_{3},\;C_{0}^{V}>0$. Therefore, the voltage consensus is achieved only, if the communication graph is connected. Furthermore, the characteristic equation of the closed-loop system, that is, $\Delta \left(s\right)=det(sI+C_{3}D^{-1}L_{d}\left(s\right)+C_{0}^{V}D_{0}D_{d}\left(s\right))=0$ has no roots in the right-half of the complex plane.

## 5 Simulation results and discussion

**Fig 2:**
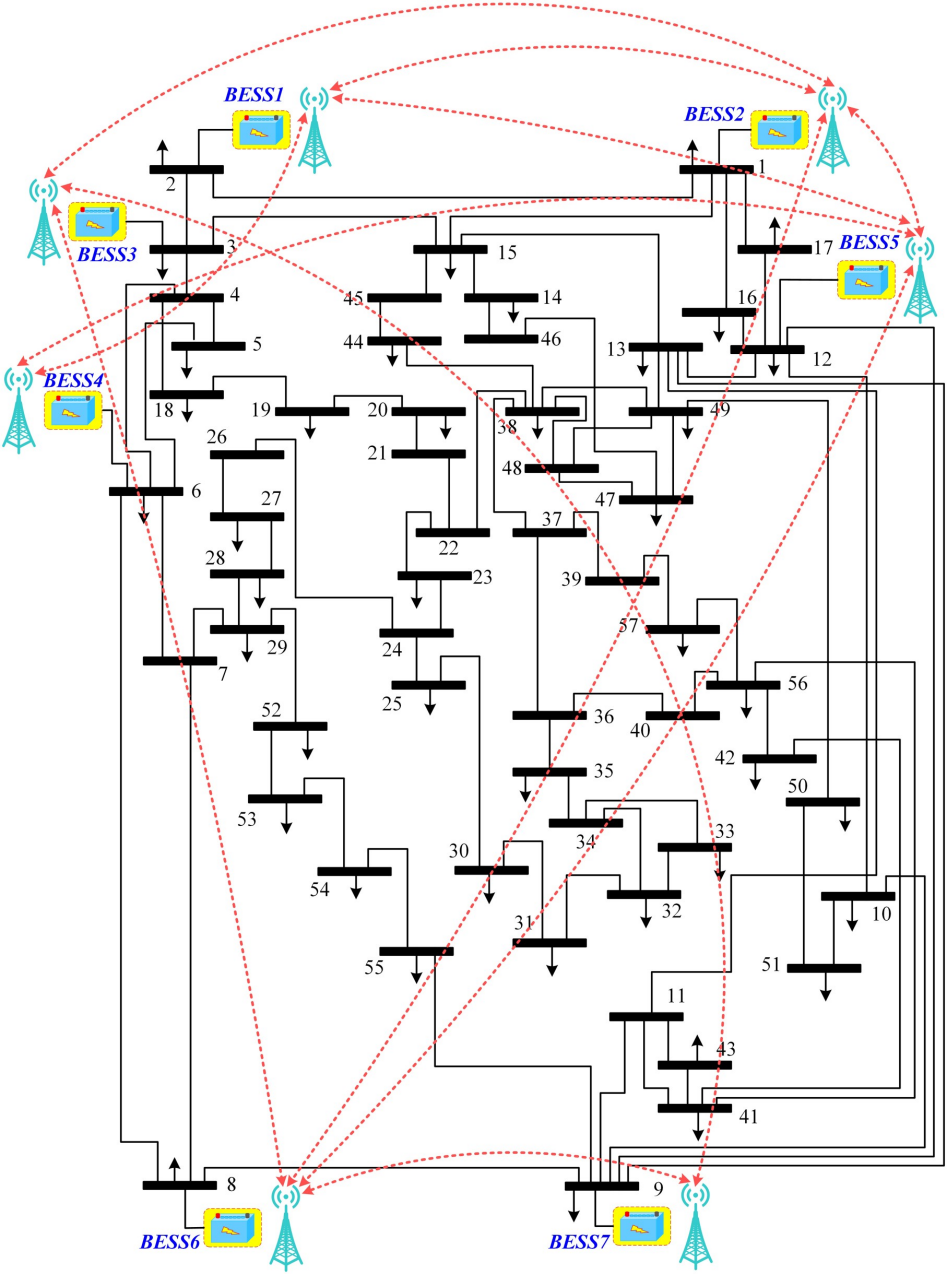
Modified IEEE 57-bus system.

**Fig 3:**
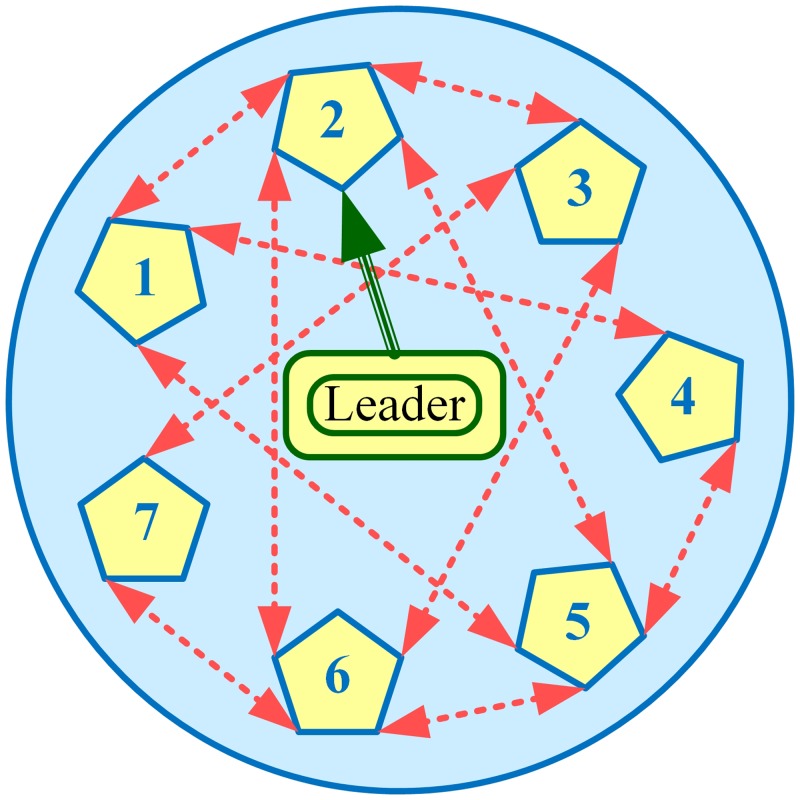
Communication graph of the BESSs.

In this section, performance of the various algorithms, described in Section 3.4, have been tested in Matlab/Simulink, through extensive simulations carried out on a modified IEEE 57-bus system with generators replaced by BESSs, as shown in Fig 2. That is, the original IEEE 57-bus system (comprising 7 generators and 42 loads) has been modified by replacing generators with BESSs. The communication graph for these 7 BESSs, that is *N* = 7 and *i* = {1, 2, 3, 4, 5, 6, 7}, with two-way communication links (represented by double-headed dotted arrows) considered in this research, is depicted in Fig 3, where BESS2 is serving as the virtual leader node to provide frequency and voltage references to other BESSs (followers). All the 7 BESSs are forced to track their (frequency and voltage) references dictated by the virtual leader node, provided that the communication graph among the BESSs is connected.

Based on the graph theory explanation given in Section 3.1, it can be seen that the communication graph, depicted in Fig 3, is undirected. The corresponding laplacian matrix for the communication graph depicted in Fig 3 (under normal conditions), is given as follows:

$$\underset{\begin{array}{c} \text{Laplacian}\;\text{matrix}\;\text{uder}\;\text{normal}\;\text{conditions}\\ \left(\text{i}.\text{e}.\;\text{without}\;\text{plug-and-play}\;\text{and}\;\text{communication}\;\text{link}\;\text{failure}\;\text{events}\right)\\ \text{where}:\;d_{i}^{in}=\{\text{3},\text{4},\text{3},\text{2},\text{4},\text{4},\text{2}\} \end{array}}{\underset{︸}{\left[\begin{array}{ccccccc} 3 & -1 & 0 & -1 & -1 & 0 & 0\\ -1 & 4 & -1 & 0 & -1 & -1 & 0\\ 0 & -1 & 3 & 0 & 0 & -1 & -1\\ -1 & 0 & 0 & 2 & -1 & 0 & 0\\ -1 & -1 & 0 & -1 & 4 & -1 & 0\\ 0 & -1 & -1 & 0 & -1 & 4 & -1\\ 0 & 0 & -1 & 0 & 0 & -1 & 2 \end{array}\right]}}$$

The performance of the proposed control strategy is tested under four different cases, described below:

**Case 1**: Analysis without plug-and-play and communication link failure events
**Case 2**: Analysis under plug-and-play and communication link failure events
**Case 3**: Switching between leader-follower and leaderless consensus without plug-and-play and communication link failure events
**Case 4**: Switching between leader-follower and leaderless consensus under plug-and-play and communication link failure events
**Case 5**: Distributed PI-based conventional control scheme without plug-and-play and communication link failure events
**Case 6**: Distributed PI-based conventional control scheme under plug-and-play and communication link failure events

***Note***: The total simulation time during each case, stated above, has been kept 24 seconds. However, to make the SoC trajectory tracking control and the ELD meaningful, the time-axis has been scaled up. Such that, on horizontal time-axis, 1 second = 1 hour. In all the remaining simulations, no scaling has been considered.

### 5.1.1 Case 1: Analysis without plug-and-play and communication link failure events

In case 1, SoC trajectory tracking control, frequency and voltage regulation, proportional active and reactive power sharing and ELD have been tested under normal conditions, that is, without plug-and-play and communication link failure events. An intentional self time-delay (of *T*_*ij*_ = 5 *ms*) and a communication time-delay (of *τ*_*ij*_ = 15 *ms*) have been considered, while carrying out analysis under case 1, to validate the robustness of the proposed strategy to communication latency.

#### SoC trajectory tracking control of the BESSs under case 1

Among different techniques for energy management, ToU-based pricing is one of the most effective method. In this method, the load users adjust their electricity consumption behaviors in response to ToU-based pricing during both peak and off-peak hours. Such that, the users are encouraged to store more energy, by charging the ESS, during off-peak hours. On the other hand, they are urged to release the stored energy, by discharging the ESS, during peak hours. Hence, these behaviors can not only reduce the overall electricity cost, but they also smooth out the demand curve [8].

To accomplish ToU-price based SoC trajectory tracking control for BESSs, first, a ToU-based price, *p*_*ToU*_, is defined. Then, a threshold price is defined with its upper and lower limits, that is, $p_{th_{up,i}}$ and $p_{th_{low,i}}$, respectively. Now, if the ToU-based price, is greater than or equal to the upper limit of the threshold price, the SoC of each BESS is maintained at its lowest level. However, if the ToU-based price is less than or equal to the lower limit of the threshold price, the SoC of each BESS is maintained at its highest level. If none of the stated conditions apply, the SoC of each BESS is maintained at the preferred/actual level. This concept is explained in Eq (48), as follows:

$$\begin{array}{c} SoC_{i_{ref}}\left(t\right)=\{\begin{array}{c} SoC_{i}^{min},\;\text{if}\;p_{ToU}\geq p_{th_{up,i}}\\ SoC_{i}^{max},\;\text{if}\;p_{ToU}\leq p_{th_{low,i}}\\ SoC_{i}\left(t\right),\;\text{otherwise} \end{array} \end{array}$$ (48)

In Eq (48), $SoC_{i_{ref}}\left(t\right)$, $SoC_{i}^{min}$, $SoC_{i}^{max}$ and *SoC*_*i*_(*t*) are the reference, minimum, maximum and actual values of the SoCs for an *i*^*th*^ BESS.

**Fig 4:**
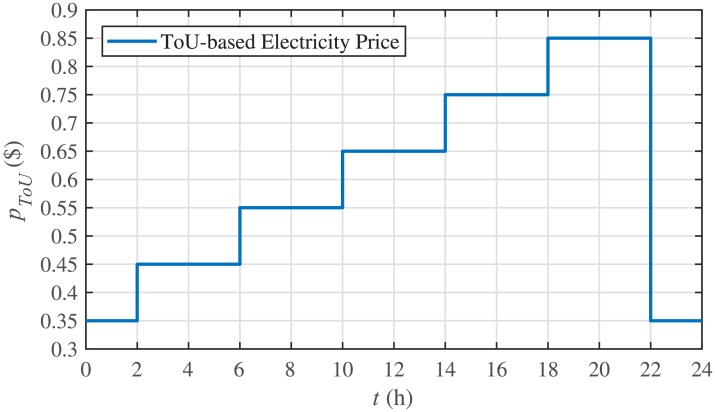
ToU-based electricity price during 24 hours.

**Fig 5:**
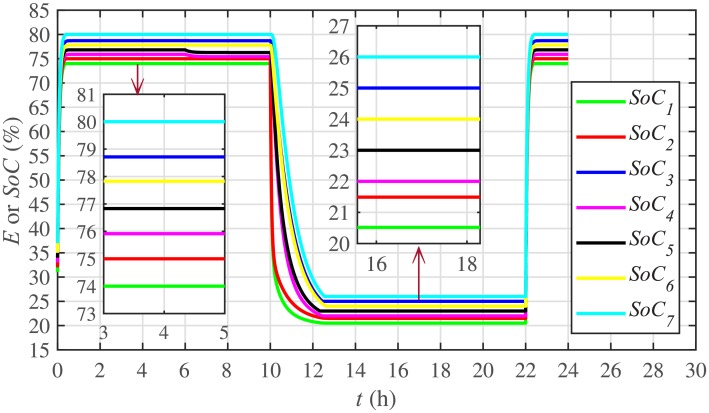
SoC tracking under case 1.

**Fig 6:**
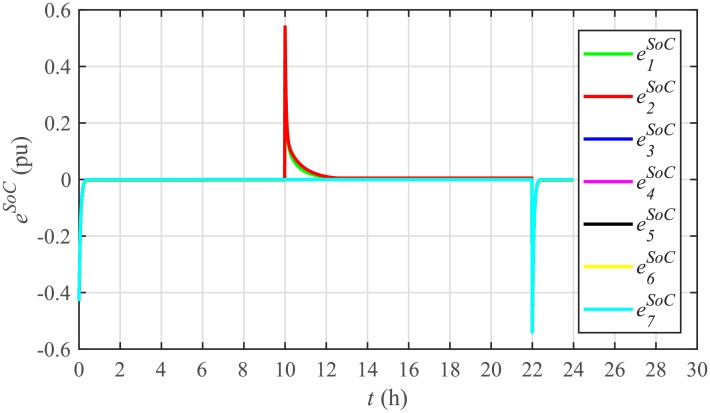
SoC tracking errors under case 1.

Different significant SoC related parameters of each BESS are given in Table 1, whereas the dynamic ToU-based electricity pricing curve for 24 hours period is illustrated in Fig 4. As depicted in Fig 4, the peak electricity price occurs during the time interval 18:00-22:00 hours, whereas, the minimum price occurs during 0:00-02:00 and 22:00-24:00 hours. The initial/present SoC values, assumed and set in the integral controllers, are indicated by *SoC*_*i*_(0) in Table 1. The SoC trajectory tracking controller parameters, expressed in Eq (9), are given in Table 1. Using Eq (48), a unique reference SoC (i.e. $E_{i_{ref}}$ or ${SoC}_{i_{ref}}$) is generated for an *i*^*th*^ BESS, based on ToU-pricing, which is then tracked by the actual SoC of the *i*^*th*^ BESS, using Eq (9). It should be noted, however, that in this particular case each BESS has its own virtual leader dictating a reference SoC, that is, $E_{i_{ref}}$ or $SoC_{i_{ref}}$, to be tracked by the actual SoC of the corresponding BESS, that is, *E*_*i*_ or *SoC*_*i*_. The SoC tracking for each BESS is illustrated in Fig 5, where each BESS starts tracking its reference/desired SoC in less than 1 hour. The zoomed-in portions of the figure validate the accurate SoC tracking with negligible steady-state error. Fig 6 shows the SoC errors, $e_{i}^{SoC}$, that is, the difference between the actual SoC and the reference SoC of an *i*^*th*^ BESS. Mathematically, it can be described as follows:

$$\begin{array}{c} e_{i}^{SoC}\left(t-T_{ij}\right)=SoC_{i}\left(t-T_{ij}\right)-SoC_{i_{ref}}=E_{i}\left(t-T_{ij}\right)-E_{i_{ref}} \end{array}$$ (49)

**Fig 7:**
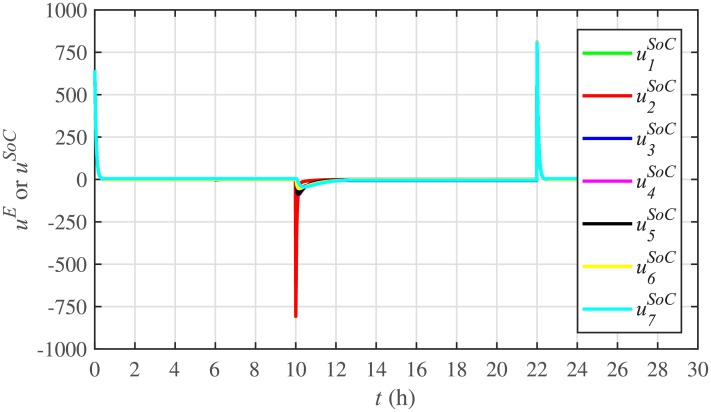
SoC tracking control efforts under case 1.

Fig 7 shows the control efforts exerted by each BESS for SoC tracking. The larger the SoC error, as shown in Fig 6, the larger will be the SoC control effort exerted, as shown in Fig 7, in the form of spikes. The spikes observed in Fig 6 at 10th and 22nd hours are due to discharging and charging power demands from the BESSs, respectively, where the SoC errors are largest. Once, all the 7 BESSs start tracking their reference SoCs, the corresponding control efforts converge to zero, implying that the system becomes stable.

**Table 1:** Different parameters of the BESSs for SoC trajectory tracking control.

BESS Index (*i*)	*SoC*_*i*_(0) (%)	$SoC_{i}^{min}\;\left(\%\right)$	$SoC_{i}^{max}\;\left(\%\right)$	$p_{th_{low,i}}\;\left(\$\right)$	$p_{th_{up,i}}\;\left(\$\right)$	$C_{1}$	$C_{0}^{E}$	$K_{i}^{P}$
1	31	20	74	0.51	0.64	6	15	0.1025
2	32	21	75	0.52	0.65	6	15	0.1017
3	36	25	79	0.56	0.69	6	15	0.0983
4	33	22	76	0.53	0.66	6	15	0.1009
5	34	23	77	0.54	0.67	6	15	0.1001
6	35	24	78	0.55	0.68	6	15	0.0992
7	37	26	80	0.57	0.70	6	15	0.0957

#### 5.1.2 Proportional active and reactive power sharing of the BESSs under case 1

For achieving proportional active power and reactive power sharing from the BESSs, the active and reactive power consensus controller parameters, expressed in Eqs (10) and (11), respectively, are given in Table 2, where *P*_*i*_(0) and *Q*_*i*_(0) indicate the initial values of powers set in the integral controllers.

**Table 2:** Different parameters of the BESSs for proportional active and reactive power sharing, and frequency and voltage regulation.

BESS Index (*i*)	$\omega_{i}^{nom}\left(0\right)\;\left(pu\right)$	$\left|V_{i}^{nom}\left(0\right)\right|\;\left(pu\right)$	*P*_*i*_(0) (*pu*)	*Q*_*i*_(0) (*pu*)	$C_{2}$	$C_{3}$	$C_{0}^{\omega }$	$C_{0}^{V}$	*α*_0*i*_	$K_{i}^{Q}$
1	2	2	6.05	10.07	6	10	10	10	1	0.1
2	1	1	7.07	5.20	6	10	0	0	0	0.121
3	2	2	10.93	3.15	6	10	10	10	1	0.125
4	2	2	8.70	9.66	6	10	0	0	0	0.113
5	2	2	9.81	6.18	6	10	10	10	1	0.098
6	2	2	9.01	4.03	6	10	10	10	1	0.108
7	2	2	10.88	7.68	6	10	0	0	0	0.114

**Fig 8:**
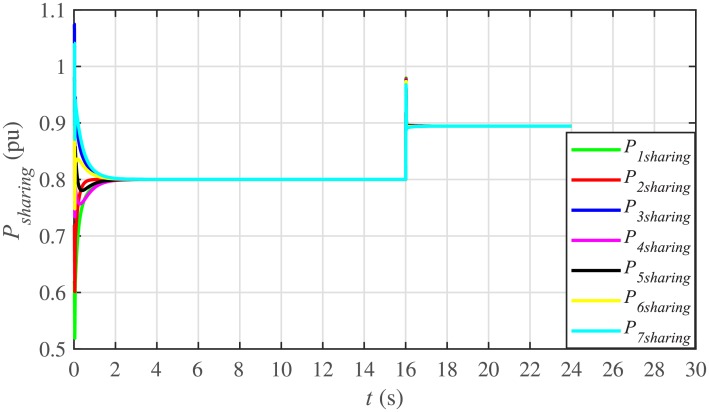
Active power sharing under case 1.

**Fig 9:**
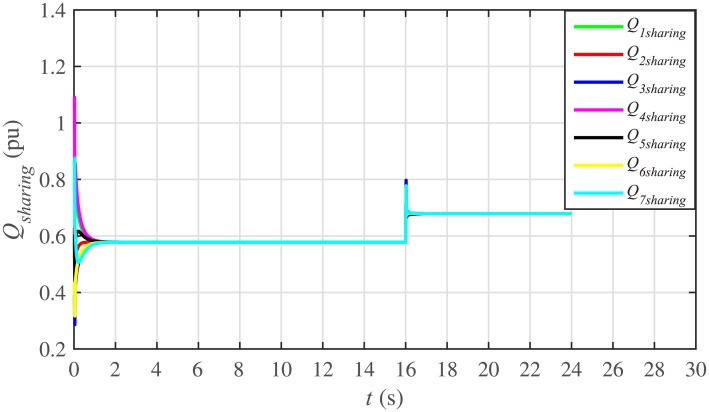
Reactive power sharing under case 1.

It is evident from Figs 8 and 9 that the consensus of active and reactive power sharing is achieved in around 2 seconds. Both active and reactive power sharing remain synchronized until there is a sudden load change (increase) of (1 + *j*1 *pu*)/BESS at the 16th second. After the load change event, both the active and reactive power sharing are successfully re-synchronized to a new (higher) value. This proves that the proposed strategy is effective for offering active and reactive power sharing control.

**Remark 1** *An important feature of the consensus control scheme is that the sum of the individual control inputs of all the BESSs is always zero. It means that the sum of the power orders of all the BESSs should be constant. The main function of the consensus control is to adjust the power output of each BESS while maintaining the total power constant. This function can also be combined with the secondary frequency and voltage control. Such that, whenever there is a load change, the secondary frequency control will handle it by adjusting the active power order of each BESS. In this way, the consensus control maintains the sum of the active power orders by adjusting the individual active power orders of the BESSs for achieving consensus. The same is true for the secondary voltage control by adjusting the reactive power orders of the BESSs*.

**Fig 10:**
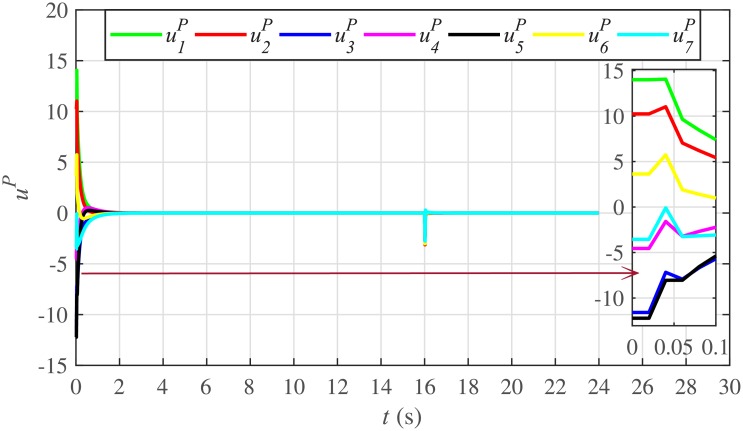
Active power control efforts under case 1.

**Fig 11:**
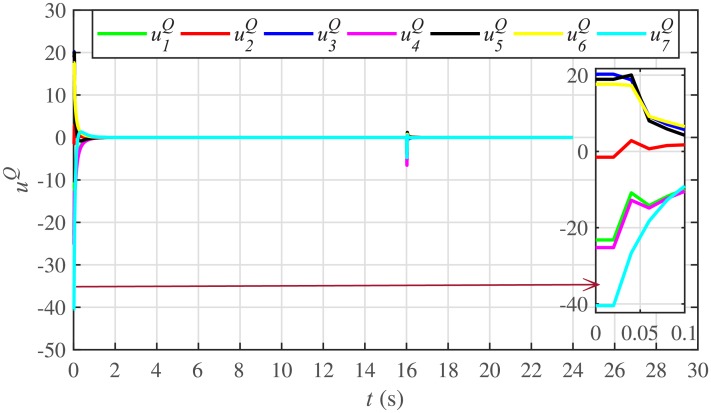
Reactive power control efforts under case 1.

Figs 10 and 11 illustrate the active and reactive power consensus control efforts, respectively. The load change event appears as transient disturbances in the control efforts. Once the active and reactive powers are synchronized, before and after the load change, the control efforts converge to zero. Thus, guaranteeing the stability of the system.

#### 5.1.3 Frequency and voltage regulation of the BESSs under case 1

**Fig 12:**
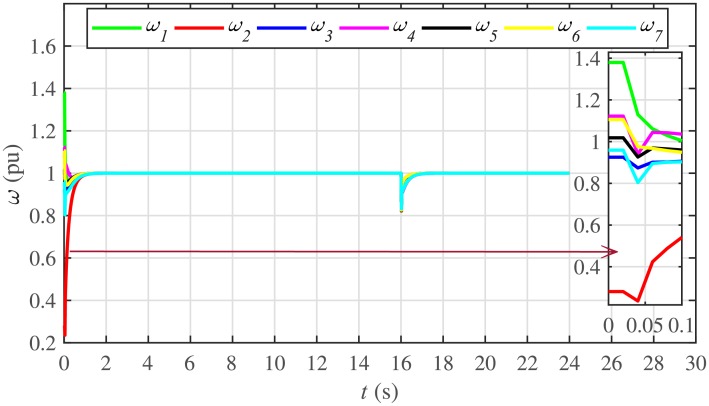
Frequency regulation under case 1.

**Fig 13:**
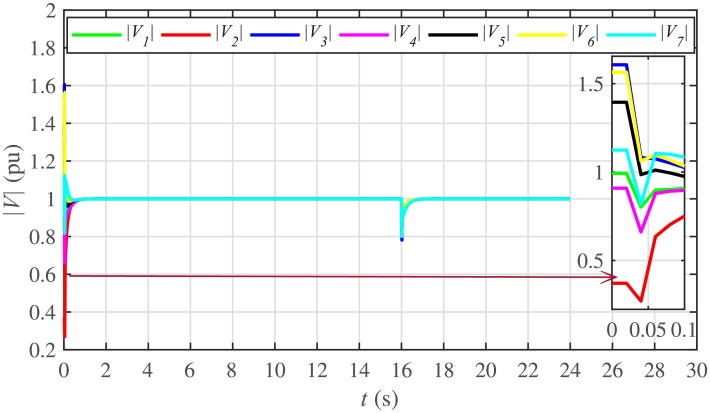
Voltage regulation under case 1.

The proposed work also supports frequency and voltage regulation of the BESSs using the leader-follower consensus approach. The frequency and voltage consensus controller parameters, expressed in Eqs (12) and (14), respectively, are given in Table 2, where $\omega_{i}^{nom}\left(0\right)$ and $\left|V_{i}^{nom}\left(0\right)\right|$ indicate the initial values for the integral controllers. For both the frequency and voltage regulation, BESS2 is set as a virtual leader node with its nominal frequency and voltage magnitude both set to 1 pu (i.e. $\omega_{2}^{nom}\left(0\right)=\omega_{ref}=1$ pu and $\left|V_{2}^{nom}\left(0\right)\right|=\left|V_{ref}\right|=1$ pu). The leader node commands/dictates its frequency and voltage magnitude and forces all the other nodes in the communications network to follow it. Hence, the frequencies and voltage magnitudes of all the 7 BESSs are regulated to *ω*_*ref*_ and |*V*_*ref*_|, respectively. The frequency and voltage tracking for the BESSs are shown in Figs 12 and 13, respectively. Note that the sudden load increase event at the 16th second appears as transient disturbances in both the frequency and voltage magnitude plots.

**Fig 14:**
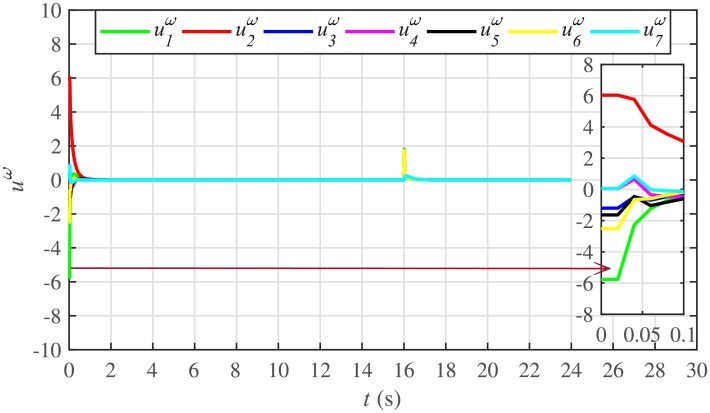
Frequency regulation control efforts under case 1.

**Fig 15:**
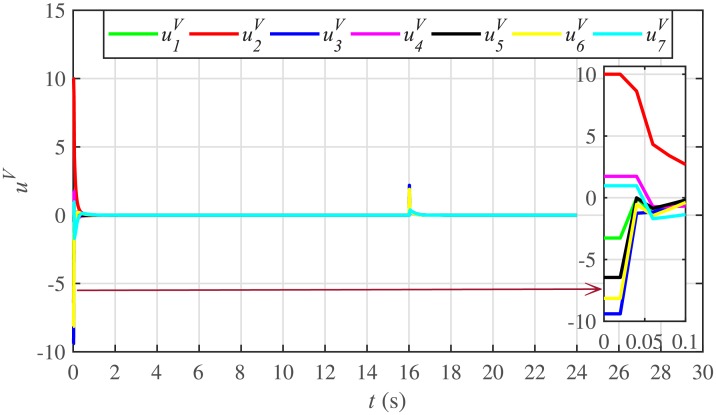
Voltage regulation control efforts under case 1.

The corresponding control efforts for frequency and voltage regulation are depicted in Figs 14 and 15, respectively. Since, both are converging to zero, once the consensuses on reference values are achieved, it implies the system is becoming stable eventually.

A slight oscillation in the vicinity of the vertical axis, depicted in the zoomed-in views, in both the frequency and voltage plots in Figs 12 and 13, respectively, is due to the communication delays. In the proposed strategy, the system is successfully tolerating a communication latency, *τ*_*ij*_, of upto 15 *ms*.

#### 5.1.4 Economic load dispatch under case 1

On generation side, the ELD is an optimization problem that aims to allocate power generation among various generating units, subject to several physical constraints, so that the system may be able to supply its load fully and most economically (with the minimum operating/generation cost) [25].

Assume *C*_*i*_(*P*_*i*,*sharing*_) to be a quadratic generation cost function for an *i*^*th*^ BESS, expressed as follows:

$$\begin{array}{c} C_{i}\left(P_{i,sharing}\right)=a_{i}P_{i,sharing}^{2}+b_{i}P_{i,sharing} \end{array}$$ (50)

where *a*_*i*_, *b*_*i*_ > 0 are the cost parameters/coefficients.

The corresponding incremental cost, λ_*i*_, for an *i*^*th*^ BESS can be expressed as follows:

$$\begin{array}{c} \lambda_{i}=\frac{dC_{i}\left(P_{i,sharing}\right)}{dP_{i,sharing}}=2a_{i}P_{i,sharing}+b_{i} \end{array}$$ (51)

Now, the total production cost, $C_{p_{i}}$, for an *i*^*th*^ BESS can be given as follows:

$$\begin{array}{c} C_{p_{i}}=\lambda_{i}\times P_{i,sharing} \end{array}$$ (52)

To achieve ELD, first of all the incremental cost, λ_*i*_, is computed using Eq (51) for an *i*^*th*^ BESS. Different values of *a*_*i*_ and *b*_*i*_, and the lower and upper limits of the active power sharing (i.e. ${\underline{P}}_{i,sharing}$ and ${\bar{P}}_{i,sharing}$, respectively, for an *i*^*th*^ BESS) and the total power demand from the system, $P_{T_{D}}$, are given in Table 3.

**Table 3:** Different parameters of the BESSs for economic load dispatch.

BESS Index (*i*)	*a*_*i*_	*b*_*i*_	$C_{4}$	${\underline{P}}_{i,sharing}\;\left(pu\right)$	${\bar{P}}_{i,sharing}\;\left(pu\right)$	$P_{T_{D}}\;\left(pu\right)$ (0 < *t* < 16) *h*	$P_{T_{D}}\;\left(pu\right)$ (16 ≤ *t* < 24) *h*
1	0.0031	0.996	0.01	0	1	5.60	6.27
2	0.0035	0.995	0.01	0	1		
3	0.0033	0.994	0.01	0	1		
4	0.0037	0.993	0.01	0	1		
5	0.0032	0.994	0.01	0	1		
6	0.0041	0.992	0.01	0	1		
7	0.0042	0.992	0.01	0	1		

**Fig 16:**
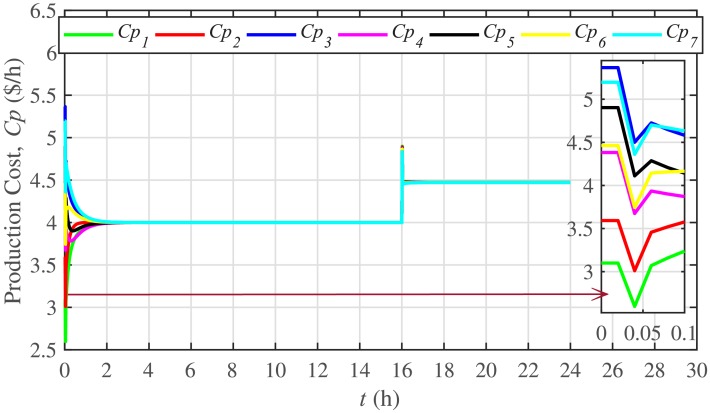
Production costs consensus under case 1.

**Fig 17:**
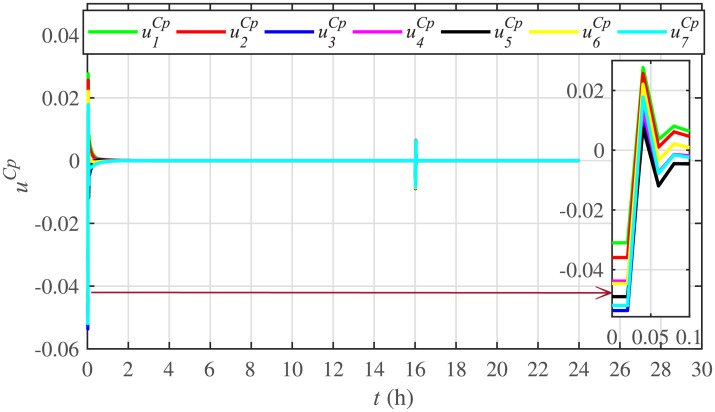
Production costs control efforts under case 1.

The ELD is ensured using Eq (16), such that, all the 7 BESSs develop consensus by synchronizing their production costs, as shown in Fig 16. Despite the load change (increase) event at the 16th hour, the production costs consensus is re-established at a new (higher) value, thus guaranteeing the effectiveness of the proposed approach. The convergence of production cost consensus control effort to zero, illustrated in Fig 17, implies the system is becoming stable.

A slight oscillation is observed in the vicinity of the vertical axis, as depicted in the zoomed-in views, in Figs 16 and 17, respectively. This is due to the communication delays. However, the system is resilient to withstand a communication latency of upto 15 *ms*.

The active power sharing mismatch, Δ*P*_*i*,*sharing*_, for an *i*^*th*^ BESS, can be expressed as follows:

$$\begin{array}{c} \Delta P_{mis}=\sum_{i=1}^{N}P_{i,sharing}-P_{T_{D}} \end{array}$$ (53)

**Fig 18:**
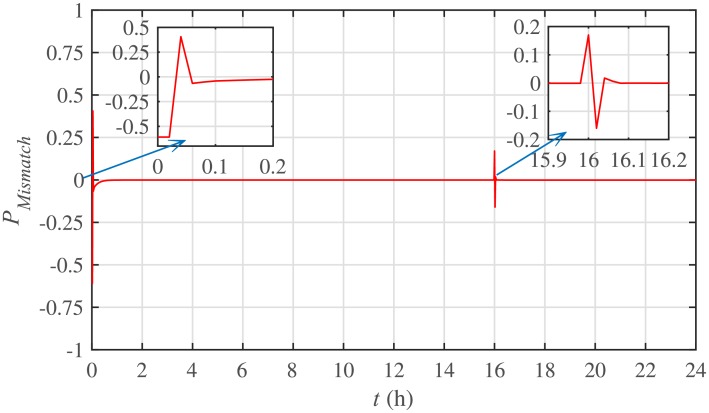
Power mismatch under case 1.

**Fig 19:**
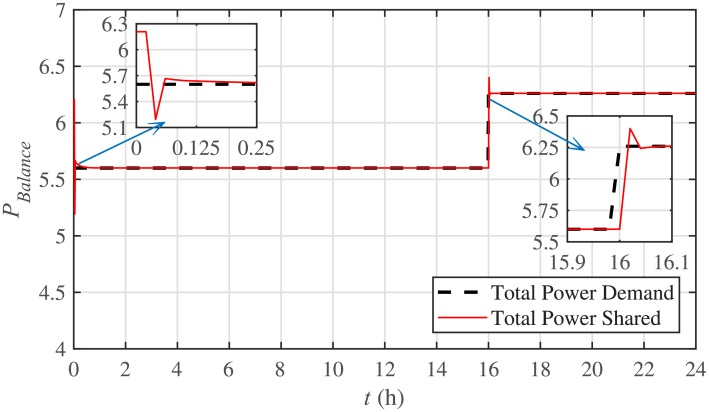
Power balance under case 1.

The power mismatch as well as the power balance are demonstrated in Figs 18 and 19, respectively. It can be seen that the system accurately fulfills the active load demand, that’s why the power mismatch converges to almost zero.

### 5.2 Case 2: Analysis under plug-and-play and communication link failure events

In case 2, SoC trajectory tracking control, frequency and voltage regulation, proportional active and reactive power sharing and ELD have been tested under plug-and-play and communication link failure events. The self time-delay and the communication time-delay have been kept the same, as discussed in case 1 (i.e. *T* = 5 *ms* and *τ* = 15 *ms*). Moreover, all the system parameters are the same as given in Tables 1, 2 and 3.

An advantage of the MAS based control architecture is to support the plug-and-play feature of the agents. To realize the plug-and-play feature, the system must be able to reconfigure/adapt itself to the alteration of communication topology (i.e. either due to disconnection/removal of an existing agent from the system, or due to integration/addition of a new agent to the system) for maintaining a seamless operation [26].

**Fig 20:**
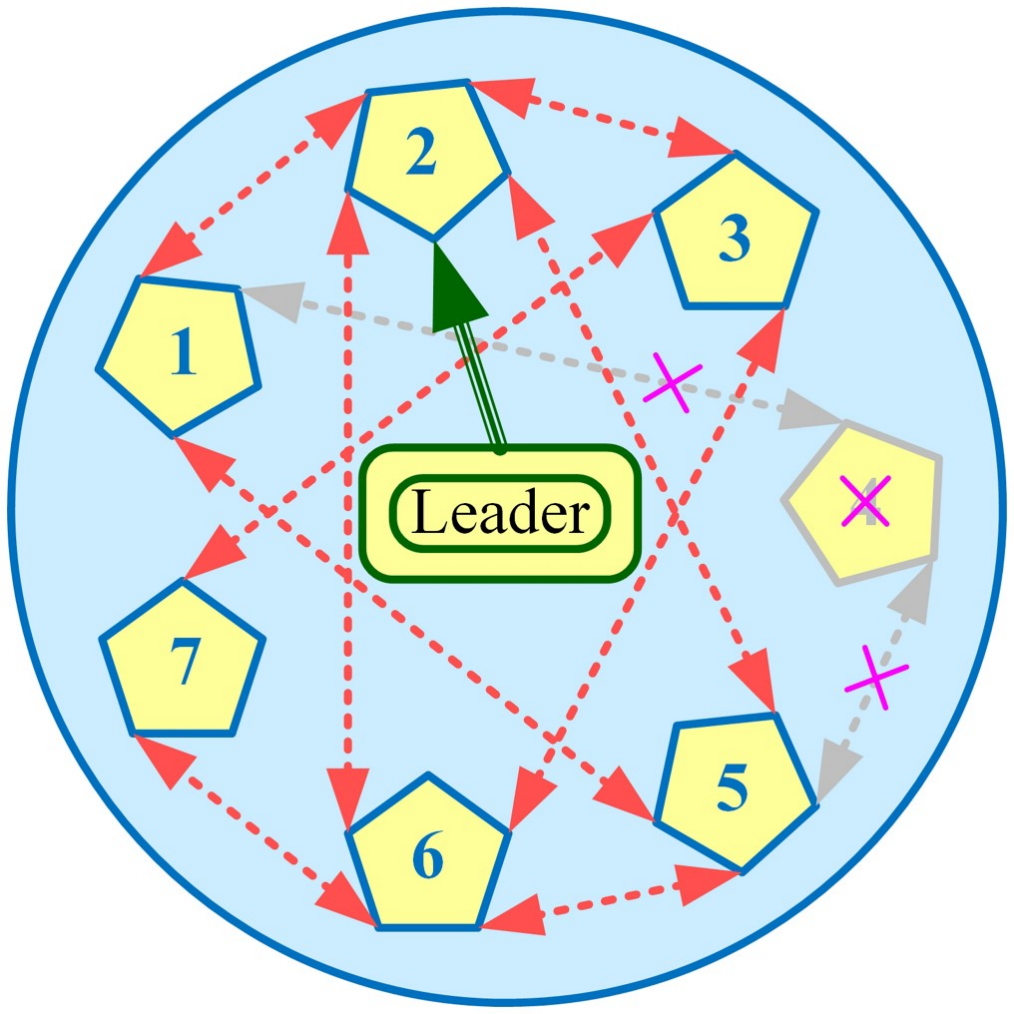
Communication graph when BESS4 is taken out of service.

**Fig 21:**
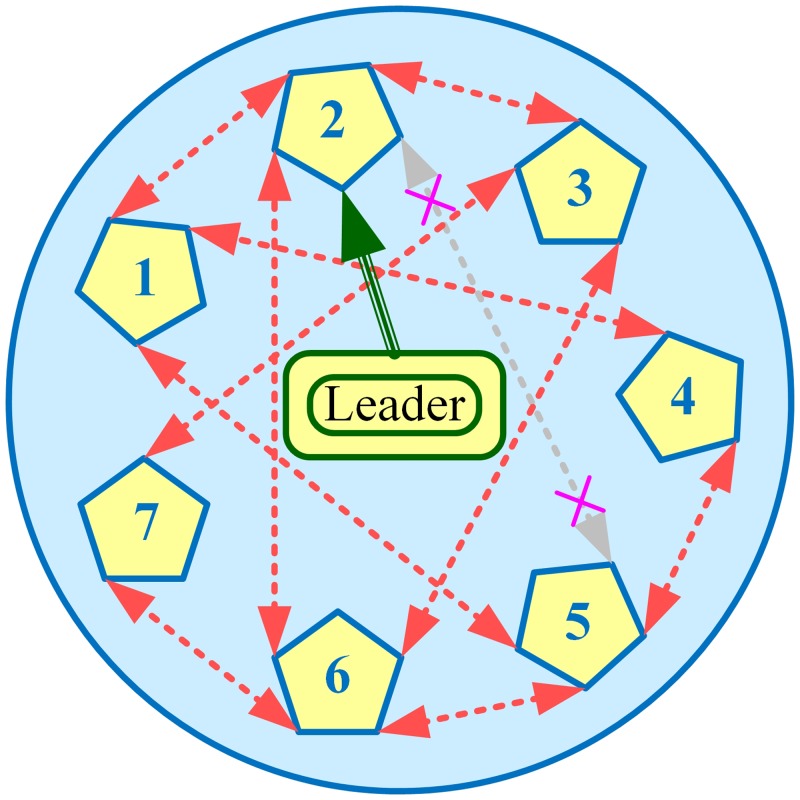
Communication graph when communication link between BESS2 and BESS5 fails.

For testing the plug-and-play feature, the BESS4 has been plugged-out at the 3rd hour and plugged-in again at the 5th hour. Similarly, for testing the robustness against communication link failure, the two-way communication link between BESS2 and BESS5 is interrupted from 10th to 12th hour. The updated communication graphs, when BESS4 is taken out of service, and communication link between BESS2 and BESS5 is interrupted, are illustrated in Figs 20 and 21, respectively.

The updated laplacian matrices, with updated entries highlighted in light violet color, when the BESS4 is taken out of service, or the two-way communication link between BESS2 and BESS5 fails, are given as follows:

$$\underset{\begin{array}{c} \text{Laplacian}\;\text{matrix}\;\text{under}\;\text{plug}-\text{and}-\text{play}\;\text{event}\\ \text{where}\;d_{i}^{in}=\{2,4,3,0,3,4,2\} \end{array}}{\underset{︸}{\left[\begin{array}{ccccccc} 2 & -1 & 0 & 0 & -1 & 0 & 0\\ -1 & 4 & -1 & 0 & -1 & -1 & 0\\ 0 & -1 & 3 & 0 & 0 & -1 & -1\\ 0 & 0 & 0 & 0 & 0 & 0 & 0\\ -1 & -1 & 0 & 0 & 3 & -1 & 0\\ 0 & -1 & -1 & 0 & -1 & 4 & -1\\ 0 & 0 & -1 & 0 & 0 & -1 & 2 \end{array}\right]}}\underset{\begin{array}{c} \text{Laplacian}\;\text{matrix}\;\text{under}\;\text{communication}\\ \text{link}\;\text{failure}\;\text{event}\\ \text{where}:\;d_{i}^{in}=\{3,3,3,2,3,4,2\} \end{array}}{\underset{︸}{\left[\begin{array}{ccccccc} 3 & -1 & 0 & -1 & -1 & 0 & 0\\ -1 & 3 & -1 & 0 & 0 & -1 & 0\\ 0 & -1 & 3 & 0 & 0 & -1 & -1\\ -1 & 0 & 0 & 2 & -1 & 0 & 0\\ -1 & 0 & 0 & -1 & 3 & -1 & 0\\ 0 & -1 & -1 & 0 & -1 & 4 & -1\\ 0 & 0 & -1 & 0 & 0 & -1 & 2 \end{array}\right]}}$$

It should be noted, however, that plugging-out the BESS4 also disables the two-way communication links between the BESS4 and the BESS1, and between the BESS4 and the BESS5, simultaneously, as clearly depicted in Fig 20.

#### 5.2.1 SoC trajectory tracking control of the BESSs under case 2

**Fig 22:**
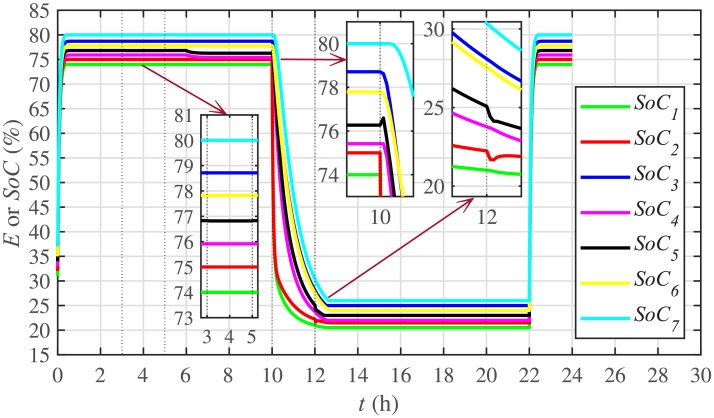
SoC tracking under case 2.

In case 2, the SoC trajectory tracking control performance is illustrated in Fig 22 for each BESS. It is evident that the SoC of the BESS4, after disconnection at the 3rd hour, remains unchanged (remains at the reference SoC value tracked before the initiation of the 3rd hour). Because, it is no more fulfilling its active and reactive load demands between the 3rd and the 5th hour. During this stated time, the active and reactive load demand of the BESS4 is shared, and hence fulfilled, by the remaining 6 BESSs. The disconnection of the BESS4 has no effect on the remaining 6 BESSs and they still tend to track their reference SoCs. When the BESS4 is reconnected at the 5th hour, it again starts tracking its most recent reference SoC.

**Fig 23:**
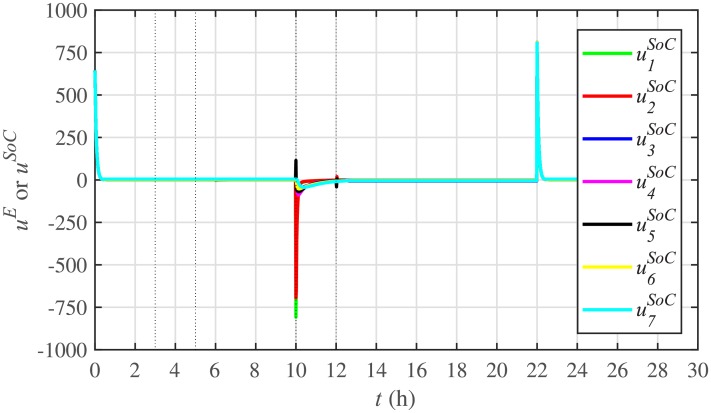
SoC tracking control efforts under case 2.

The effect of communication link failure event between the BESS2 and the BESS5, from 10th to 12th hour, on the SoC tracking performance is depicted in Fig 22, in the form of very small excursions, especially in the vicinity of the 12th hour. However, the proposed algorithm has the ability to fast correct the SoC values. This implies that the system is also robust against two-way communication link failure between the BESSs. The plug-and-play event is almost unnoticeable in the SoC tracking control efforts, illustrated in Fig 23. However, minor disturbances can be seen for the communication link failure event.

#### 5.2.2 Proportional active and reactive power sharing of the BESSs under case 2

**Fig 24:**
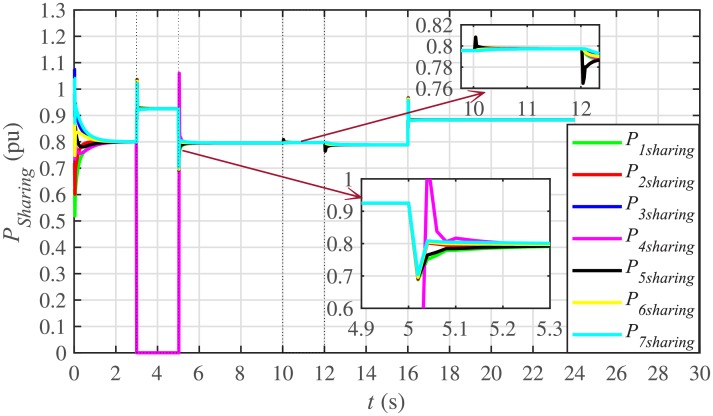
Active power sharing under case 2.

**Fig 25:**
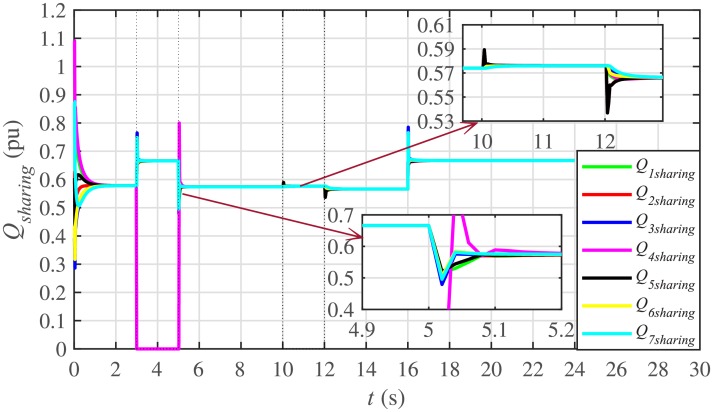
Reactive power sharing under case 2.

**Fig 26:**
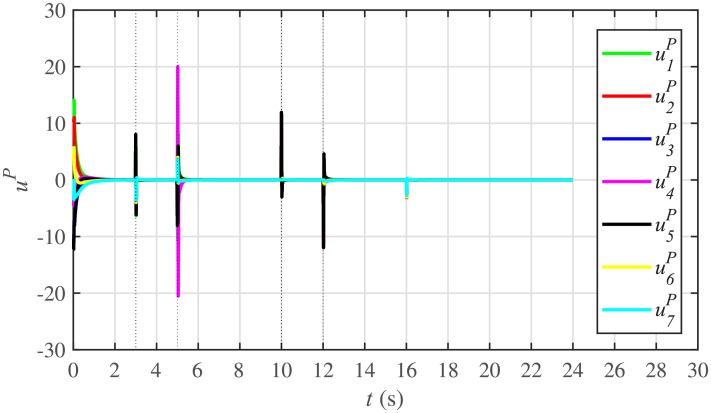
Active power control efforts under case 2.

**Fig 27:**
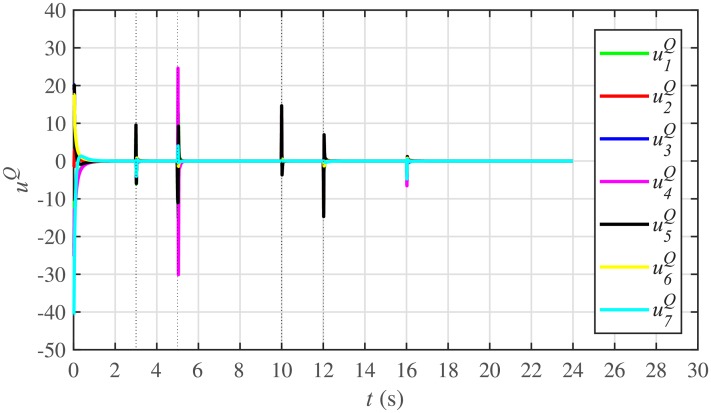
Reactive power control efforts under case 2.

In case 2, the active and reactive power sharing controls have also been tested. Taking the BESS4 out of service at the 3rd second, makes their active and reactive power sharing (i.e. *P*_4,*sharing*_ and *Q*_4,*sharing*_, respectively) equal to zero, as illustrated in Figs 24 and 25, respectively. Because, it is neither dispatching nor absorbing any power. However, during this time, the active and reactive load demand of the BESS4 is still fulfilled by the remaining 6 BESSs. For this purpose, the remaining 6 BESSs have to increase their power sharing outputs to share and support the power previously supplied by the BESS 4. This also supports the fact that during this time the SoC of the BESS4 becomes constant, as shown in Fig 22. Although, the BESS4 is disconnected, the remaining 6 BESSs still provide proportional active and reactive power sharing, because, the communication network among the stated 6 BESSs is still a connected graph. At the 5th second, when the BESS4 is reconnected, its active and reactive power sharing are re-synchronized to the system after a very fast transient. Both the plug-and-play and communication link failure events lead to minor disturbances in the system, as shown in Figs 24 and 25, featuring the synchronization of active and reactive power sharing, and Figs 26 and 27, featuring their corresponding control efforts, respectively. It implies, the proposed scheme is capable of fast correcting the regulation of active and reactive power sharing and maintaining the proper operation. Thus, the proposed strategy offers good plug-and-play capability as well as robustness against communication link failure.

#### 5.2.3 Frequency and voltage regulation of the BESSs under case 2

**Fig 28:**
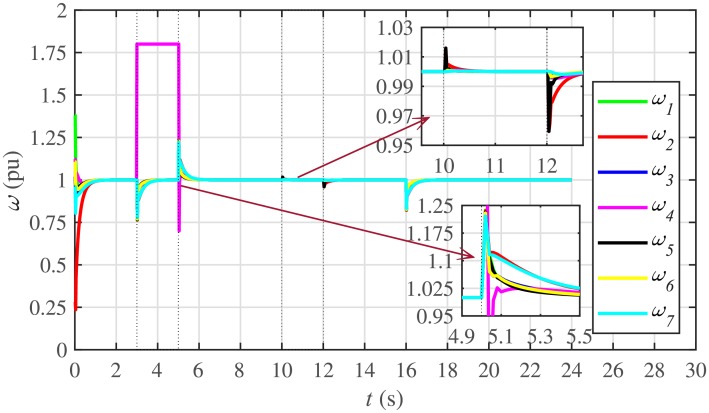
Frequency regulation under case 2.

**Fig 29:**
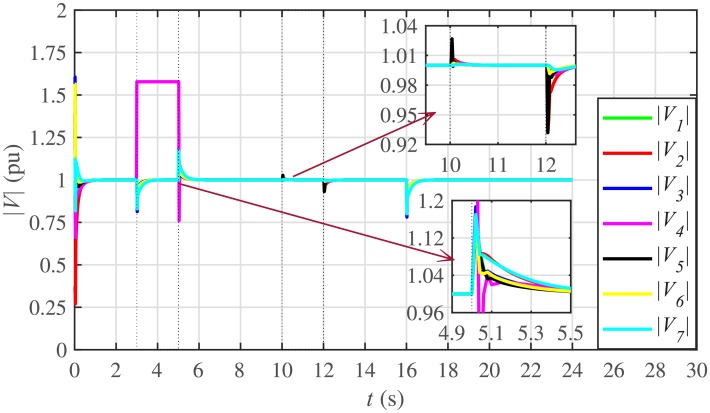
Voltage regulation under case 2.

**Fig 30:**
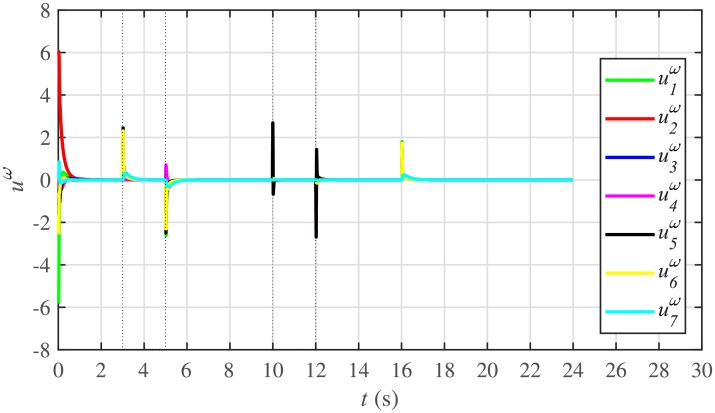
Frequency regulation control efforts under case 2.

**Fig 31:**
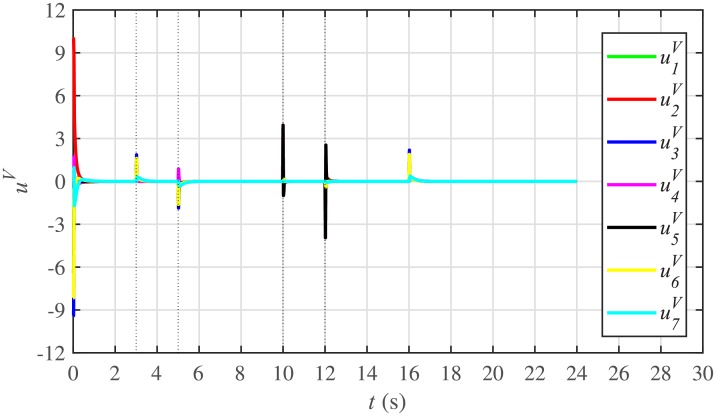
Voltage regulation control efforts under case 2.

The frequency and voltage regulation tests have also been carried out in case 2. As shown in Figs 28 and 29, when the BESS4 is unplugged at the 3rd second, its frequency and voltage (i.e. *ω*_4_ and |*V*_4_|, respectively) both increase and remain at the most recent nominal values till the 5th second. During this time, both $u_{4}^{\omega }$ and $u_{4}^{V}$ also remain zero, as depicted in Figs 30 and 31, respectively. However, the remaining 6 BESSs are still synchronizing their frequencies to the leader node. Re-plugging BESS4 at the 5th second, re-synchronizes both the frequency and voltage of the BESS4 to the system (i.e. *ω*_*ref*_ = 1 and |*V*_*ref*_| = 1, respectively) after a fast transient. Similarly, the interruption of the two-way communication link between BESS2 and BESS5 leads to minor transient disturbances in both the frequency and voltage. In other words, the proposed algorithm has the capability of fast correcting the frequency and voltage magnitude. Both the plug-and-play and communication link failure events are reflected as minor transient disturbances on the frequency and voltage regulation of the system. It can easily be concluded that the system is not only providing a good plug-and-play capability, but also robustness against communication link failure.

#### 5.2.4 Economic load dispatch for the BESSs under case 2

**Fig 32:**
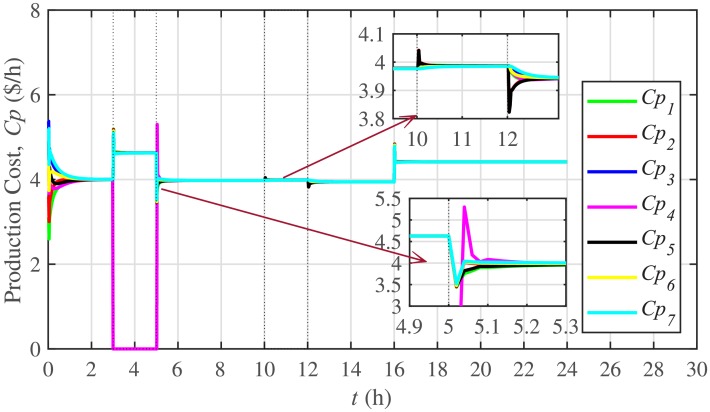
Production costs consensus under case 2.

**Fig 33:**
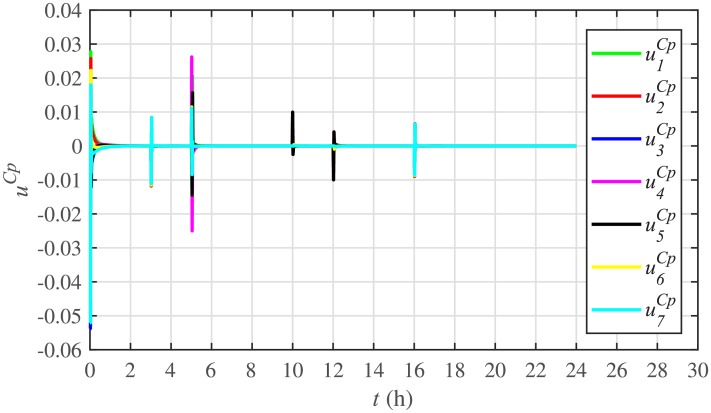
Production costs control efforts under case 2.

The ELD for the BESSs is also tested in case 2 and the results for production cost consensus and the corresponding control efforts are plotted in Figs 32 and 33, respectively. When the BESS4 is plugged-out at the 3rd hour, production cost of the BESS4 goes to zero. Because, it is not dispatching any active power. However, the load demand of the BESS4 is shared and supported by the remaining 6 BESSs, which results in a higher production cost value (due to the increase in load of the remaining 6 BESSs). Re-plugging BESS4 at the 5th hour brings the production cost of the system back to its normal value after a sharp transient. On the other hand, there is a negligible effect of the communication link failure event between BESS2 and BESS5, from 10th to 12th hour, on the production cost consensus of the system. Hence, it can easily be concluded that the system possesses both plug-and-play capability and robustness against communication link failure.

**Fig 34:**
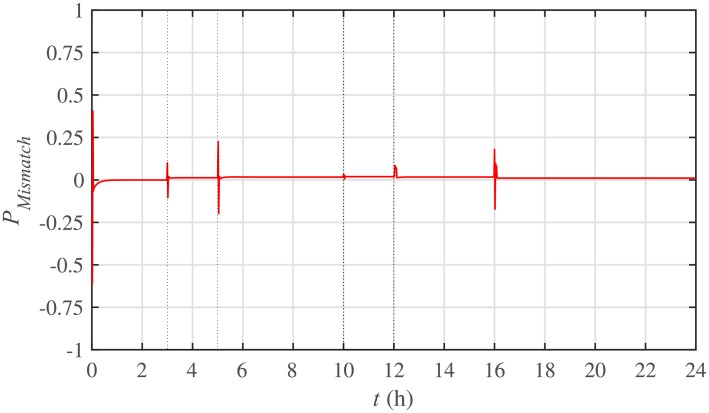
Power mismatch under case 2.

**Fig 35:**
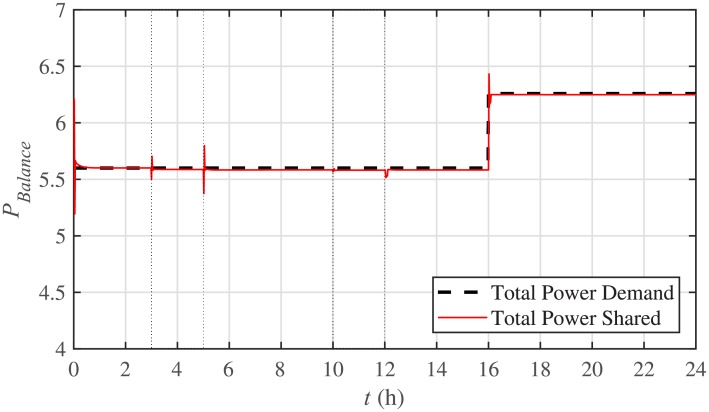
Power balance under case 2.

The corresponding power mismatch as well as the power balance, in case 2, are illustrated in Figs 34 and 35, respectively. It can be seen that the system accurately fulfills the active load demand, that’s why, the power mismatch converges to almost zero.

### 5.3 Case 3: Switching between leader-follower and leaderless consensus without plug-and-play and communication link failure events

In this case study the proposed algorithm is tested when switching between leader-follower and leaderless consensus occurs without plug-and-play and communication link failure events. For this purpose, the leadership status of node 2 is deactivated from 15th to 20th second by setting *α*_0*i*_ = 0 in Eqs (12) and (14), while for rest of the simulation time it still acts as the leader node. The communication graph is the same as illustrated in Fig 3.

**Fig 36:**
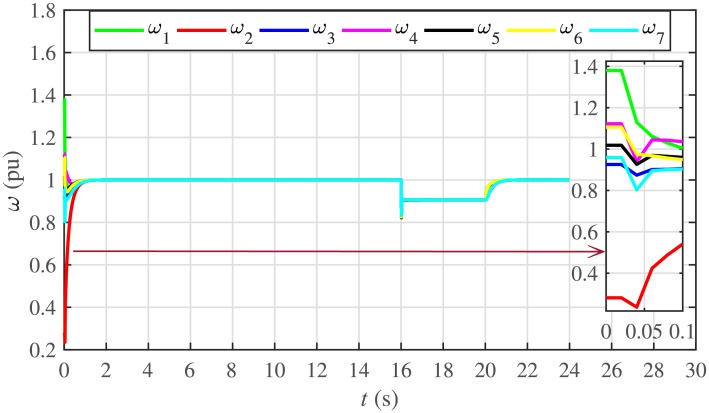
Frequency regulation with leader-follower and leaderless consensus under case 3.

**Fig 37:**
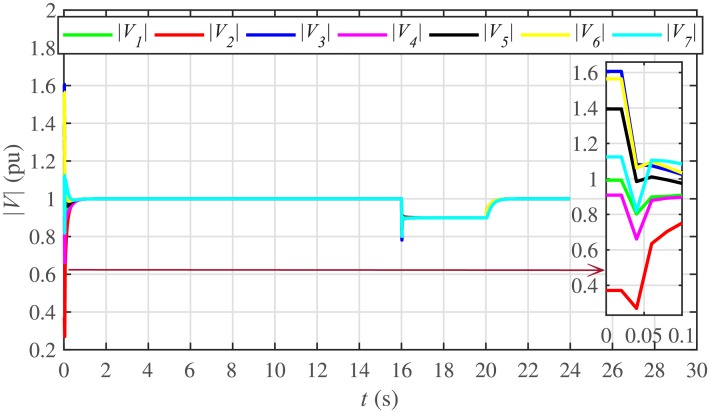
Voltage regulation with leader-follower and leaderless consensus under case 3.

The frequency and voltage consensus plots are depicted in Figs 36 and 37, respectively. It can be seen that even if the leader node 2 loses its leadership status at the onset of 15th second, the frequency and voltage magnitude still remain synchronized to *ω*_*ref*_, |*V*_*ref*_| = 1 *pu*, respectively, previously dictated by the leader node till 16th second, as long as the load remains constant. But once the load changes (increases) at 16th second, both the frequency and voltage magnitude decrease and the consensus is developed on new (lower) values. However, when the leadership status of node is restored at 20th second, both the frequency and voltage magnitude are restored to *ω*_*ref*_, |*V*_*ref*_| = 1 *pu*, respectively, after a sharp transient.

It means that the proposed algorithm can equally be applied for developing a leader-follower and leaderless consensus.

### 5.4 Case 4: Switching between leader-follower and leaderless consensus under plug-and-play and communication link failure events

In this case study the proposed algorithm is further tested when switching between leader-follower and leaderless consensus occurs in the presence of plug-and-play and communication link failure events. The communication graphs are the same as those illustrated in Figs 20 and 21.

**Fig 38:**
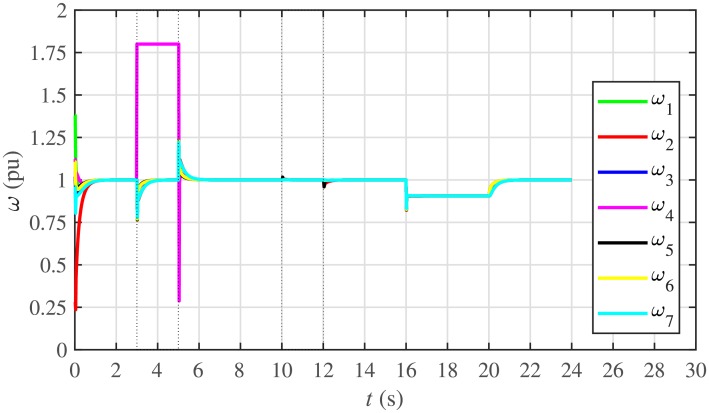
Frequency regulation with leader-follower and leaderless consensus under case 4.

**Fig 39:**
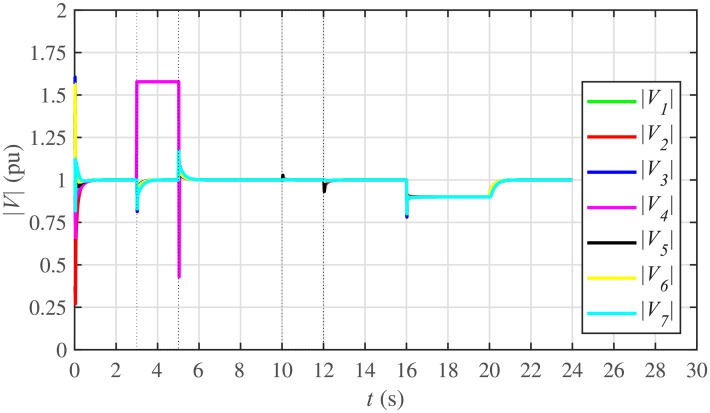
Voltage regulation with leader-follower and leaderless consensus under case 4.

The frequency and voltage consensus plots are depicted in Figs 38 and 39, respectively. It is evident that even in the presence of plug-and-play (from 3rd to 5th second) and communication link failure (from 10th to 12th second), the proposed algorithm successfully supports both the leaderless (from 15th to 20th second) and leader-follower consensus (from 0 to 15th second and 20th to 24th second).

### 5.5 Case 5: Distributed PI-based conventional control scheme without plug-and-play and communication link failure events [27]

In this section, the proposed distributed control scheme is tested against the distributed PI-based conventional control scheme, formulated in [27], without plug-and-play and communication link failure events as described in case 1.

In this stated conventional strategy, the authors have shown that for a large class of systems, the decentralized PI-control is not a feasible control strategy. Instead, they have formulated a distributed controller that mimics a decentralized P-controller with a centralized I-controller by distributed averaging. Although the proportional part of this controller is decentralized, the overall controller is distributed due to the communication needs of the distributed integral part.

Under distributed PI-based control scheme, the frequency and active power sharing control can be expressed as follows:

$$\begin{array}{c} \begin{array}{cc} & {\dot{\omega }}_{i}\left(t\right)=[\omega_{ref}-\omega_{i}\left(t-T_{ij}\right)]-\gamma \sum_{j\in N_{i}}a_{ij}[\omega_{i}\left(t-T_{ij}\right)-\omega_{j}\left(t-\tau_{ij}\right)]-\beta u_{i}^{P}\left(t-T_{ij}\right) \end{array} \end{array}$$ (54)

$$\begin{array}{c} \begin{array}{cc} & u_{i}^{P}\left(t-T_{ij}\right)=P_{i,sharing}\left(t-T_{ij}\right)=\kappa_{i}^{P}[\omega_{ref}-\omega_{i}\left(t-T_{ij}\right)]+\kappa_{i}^{I}\omega_{i}\left(t-T_{ij}\right) \end{array} \end{array}$$ (55)

where *γ*, *β* > 0, $\kappa_{i}^{P}$, $\kappa_{i}^{I}>0$ are the proportional and integral gains, respectively, and *a*_*ij*_ is the entry of the adjacency matrix.

Similarly, under distributed PI-based control scheme, the voltage and reactive power sharing control can be expressed as follows:

$$\begin{array}{c} \begin{array}{cc} & |{\dot{V}}_{i}\left(t-T_{ij}\right)|=[|V_{ref}|-\left|V_{i}\left(t-T_{ij}\right)\right|]-\Gamma \sum_{j\in N_{i}}a_{ij}[\left|V_{i}\left(t-T_{ij}\right)\right|-\left|V_{j}\left(t-\tau_{ij}\right)\right|]\\ & -\zeta u_{i}^{Q}\left(t-T_{ij}\right) \end{array} \end{array}$$ (56)

$$\begin{array}{c} \begin{array}{cc} & u_{i}^{Q}\left(t-T_{ij}\right)=Q_{i,sharing}\left(t-T_{ij}\right)=\kappa_{i}^{P}[\left|V_{ref}\right|-|V_{i}\left(t-T_{ij}\right)]+\kappa_{i}^{I}\left|V_{i}\left(t-T_{ij}\right)\right| \end{array} \end{array}$$ (57)

where Γ, *ζ* > 0.

**Fig 40:**
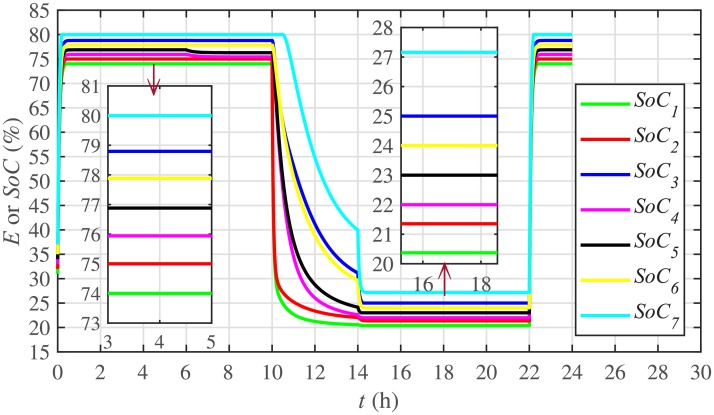
SoC tracking under case 5. [27].

**Fig 41:**
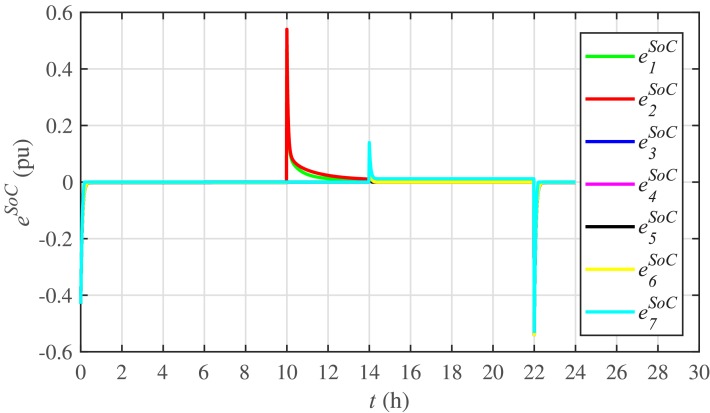
SoC tracking errors under case 5. [27].

**Fig 42:**
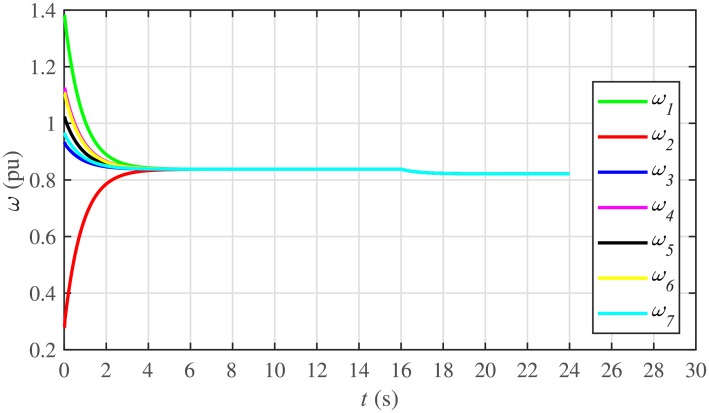
Frequency regulation under case 5. [27].

**Fig 43:**
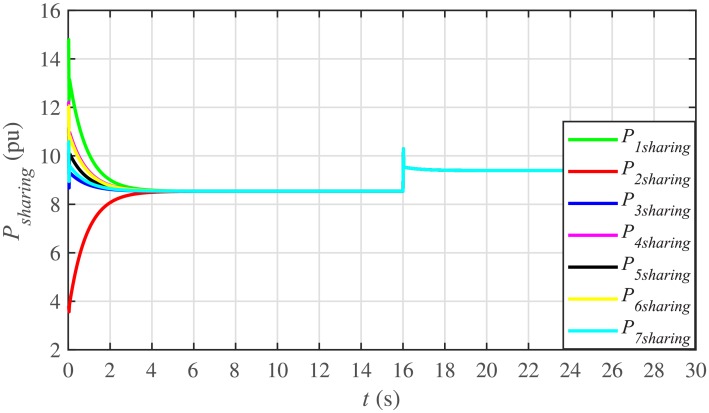
Active power sharing under case 5. [27].

**Fig 44:**
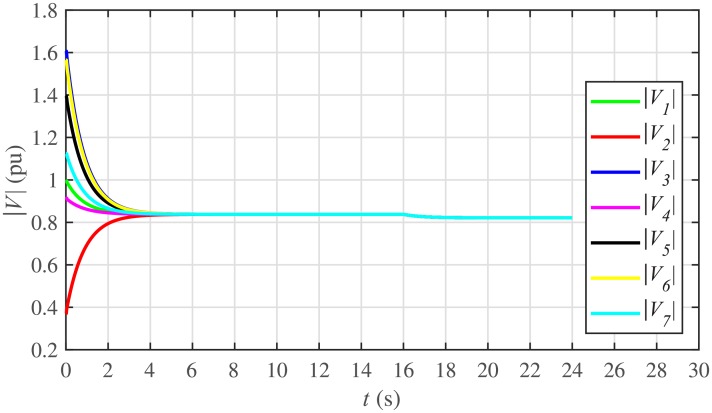
Voltage regulation under case 5. [27].

**Fig 45:**
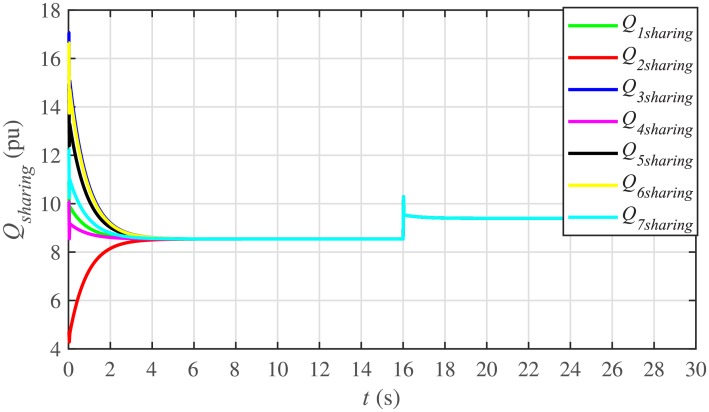
Reactive power sharing under case 5. [27].

**Fig 46:**
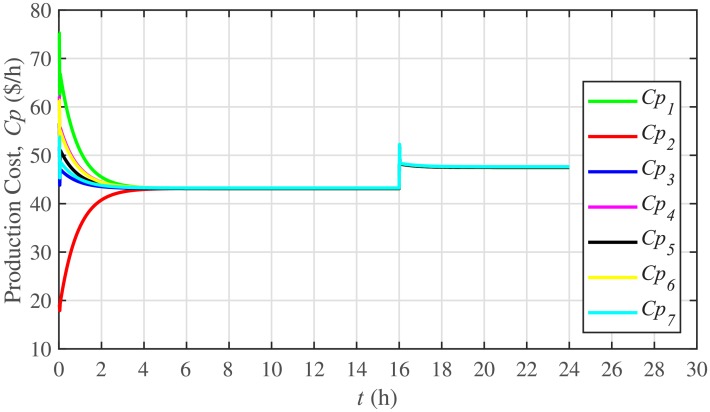
Production cost consensus under case 5. [27].

Under distributed PI-based control scheme, SoC trajectory tracking along with corresponding errors are illustrated in Figs 40 and 41, respectively. When compared with their proposed counterparts, as shown in Figs 5 and 6, one can easily conclude that the proposed scheme offers superior SoC tracking with lesser error. The frequency and corresponding active power sharing regulation under distributed PI-based control scheme are depicted in Figs 42 and 43, respectively. It is evident that the consensus development/convergence is slow (around 5 seconds) when compared with the proposed scheme (around 2 seconds), as depicted in Figs 8 and 12. Similarly, the voltage and corresponding reactive power sharing regulation under distributed PI-based control scheme are illustrated in Figs 44 and 45, respectively. Again, it can be seen that the consensus development/convergence is slow (around 5 seconds) when compared with the proposed scheme (around 2 seconds), as depicted in Figs 9 and 13. Furthermore, another major drawback of the distributed PI-based conventional control strategy is that both the frequencies and voltages of the BESSs are not exactly regulated to their reference values (*ω*_*ref*_, |*V*_*ref*_| = 1 *pu*), before and after the load change (increase) event at the 16th second. Finally, the production cost consensus (i.e. ELD) is shown in Fig 46, where the consensus development is slow, when compared with the proposed scheme as depicted in Fig 16.

Consequently, the proposed distributed control strategy is superior to the conventional PI-based distributed control strategy in terms of offering faster convergence and minor error.

### 5.6 Case 6: Distributed PI-based conventional control scheme under plug-and-play and communication link failure events [27]

In this section, the proposed distributed control scheme is further tested against the distributed PI-based conventional control scheme under plug-and-play and communication link failure events as discussed in case 2.

**Fig 47:**
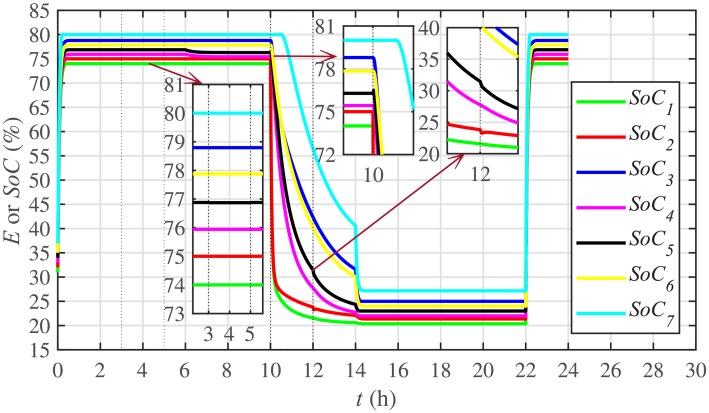
SoC tracking under case 6. [27].

**Fig 48:**
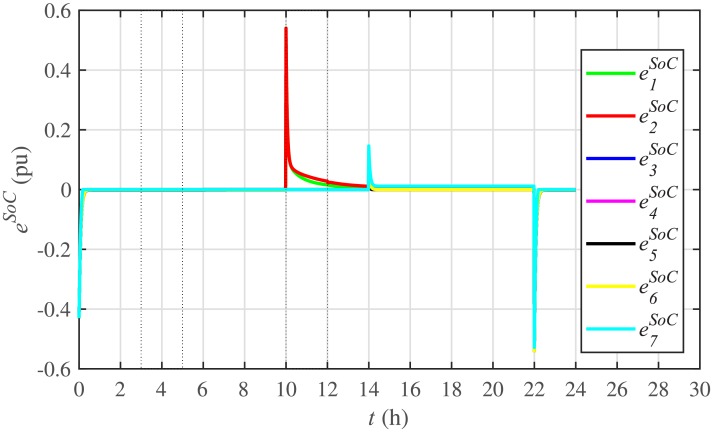
SoC tracking errors under case 6. [27].

**Fig 49:**
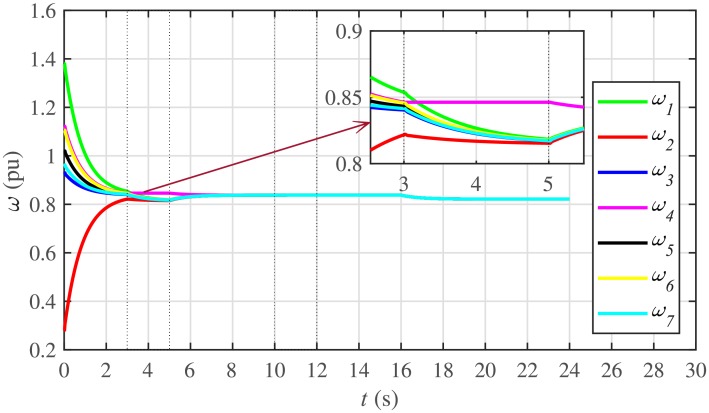
Frequency regulation under case 6. [27].

**Fig 50:**
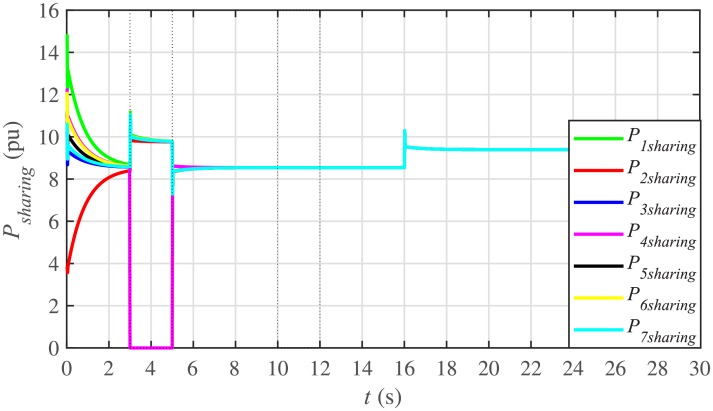
Active power sharing under case 6. [27].

**Fig 51:**
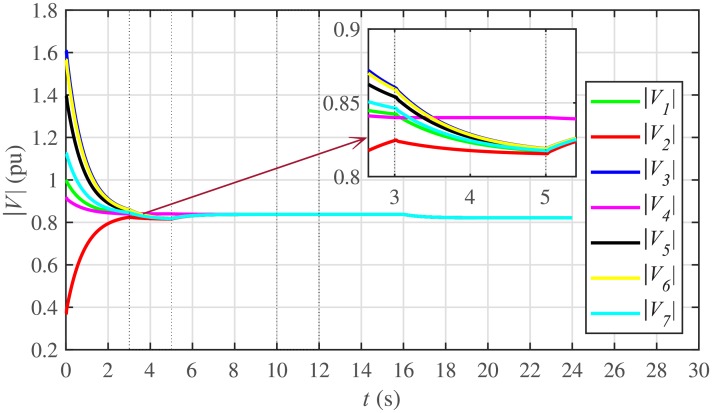
Voltage regulation under case 6. [27].

**Fig 52:**
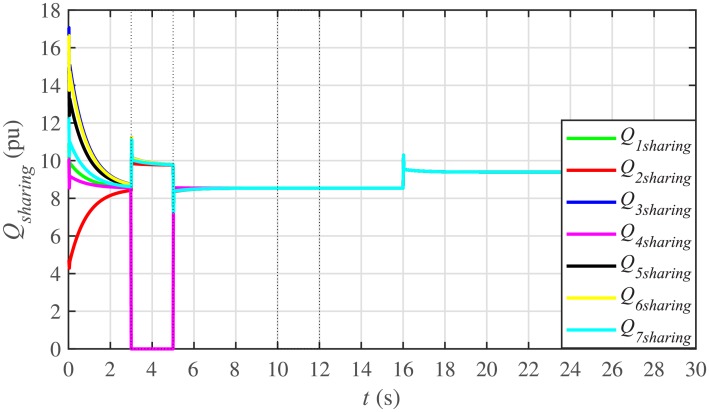
Reactive power sharing under case 6. [27].

**Fig 53:**
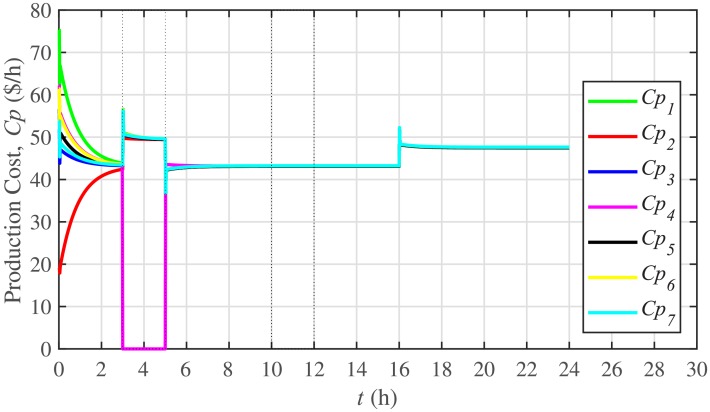
Production cost consensus under case 6. [27].

Various results under conventional distributed PI-based control strategy are shown in Figs 47–53. This scheme is also supporting the plug-and-play operation. Such that, unplugging BESS4 makes it frequency and voltage constant, having no role towards consensus development. Moreover, the active and reactive power sharing and production cost of the BESS4 become zero. One can easily observe that the convergence is slow when compared with their proposed counterparts, as depicted in Figs 22–25, 28, 29 and 32. Moreover, in this case again, the frequencies and voltages of the BESSs are not exactly regulated to their reference values. However, in the conventional scheme, the link failure and restoration event is seamless.

As a result, the proposed distributed control scheme offers a superior performance to the conventional PI-based distributed control strategy in terms of having faster convergence and lesser error.

## Conclusions

In this research article, a multi-agent consensus based distributed control algorithm is proposed for fulfilling multiple objectives, simultaneously, in a microgrid. The proposed algorithm considers the hierarchical control structure of the BESSs and the frequency/voltage droop controllers. It successfully tracks the the SoC reference trajectories of multiple BESSs, generated on time of use based pricing, for 24 hours varying load. Furthermore, it dispatches the load economically, realizes the active and reactive power synchronization, and frequency and voltage regulation (using leader-follower consensus approach), simultaneously. Each BESS only requires its own (local) information and information from the neighboring BESSs through a sparse communication network. Plug-and-play and robustness against communication link failure features are embodied in the proposed algorithm. Moreover, it is resilient to communication latency of upto 15 *ms*. The effectiveness of the proposed algorithm is tested and validated through extensive simulations carried out in Matlab/Simulink on a modified IEEE 57-bus system, where it has been found to have a very fast convergence and negligible steady-state error. The superior performance of the proposed distributed control strategy is demonstrated by comparing is performance with the distributed PI-based conventional control strategy.
